# Further analysis of barley MORC1 using a highly efficient RNA‐guided Cas9 gene‐editing system

**DOI:** 10.1111/pbi.12924

**Published:** 2018-05-07

**Authors:** Neelendra Kumar, Matteo Galli, Jana Ordon, Johannes Stuttmann, Karl‐Heinz Kogel, Jafargholi Imani

**Affiliations:** ^1^ Research Centre for BioSystems, Land Use and Nutrition Institute of Phytopathology Justus‐Liebig University Giessen Giessen Germany; ^2^ Institute of Genetics Martin Luther University of Halle‐Wittenberg Halle Saale Germany

**Keywords:** CRISPR/Cas9, gene‐editing, barley, wheat, rice, MORC1, *Blumeria*, *Fusarium*

## Abstract

Microrchidia (MORC) proteins comprise a family of proteins that have been identified in prokaryotes and eukaryotes. They are defined by two hallmark domains: a GHKL‐type ATPase and an S5‐fold. In plants, MORC proteins were first discovered in a genetic screen for *Arabidopsis thaliana* mutants compromised for resistance to a viral pathogen. Subsequent studies expanded their role in plant immunity and revealed their involvement in gene silencing and genome stabilization. Little is known about the role of MORC proteins of cereals, especially because knockout (KO) mutants were not available and assessment of loss of function relied only on RNAi strategies, which were arguable, given that MORC proteins in itself are influencing gene silencing. Here, we used a *Streptococcus pyogenes* Cas9 (*Sp*Cas9)‐mediated KO strategy to functionally study *HvMORC1*, one of the current seven *MORC* members of barley. Using a novel barley RNA Pol III‐dependent *U3* small nuclear RNA (snRNA) promoter to drive expression of the synthetic single guide RNA (sgRNA), we achieved a very high mutation frequency in *HvMORC1*. High frequencies of mutations were detectable by target sequencing in the callus, the T0 generation (77%) and T1 generation (70%–100%), which constitutes an important improvement of the gene‐editing technology in cereals. Corroborating and extending earlier findings, *Sp*Cas9‐edited *hvmorc1‐* KO barley, in clear contrast to Arabidopsis *atmorc1* mutants, had a distinct phenotype of increased disease resistance to fungal pathogens, while *morc1* mutants of either plant showed de‐repressed expression of transposable elements (TEs), substantiating that plant MORC proteins contribute to genome stabilization in monocotyledonous and dicotyledonous plants.

## Introduction

Gene‐editing methods have arisen as an efficient tool for rapid analysis of gene function. From the agricultural perspective, these new methods can be harnessed to create crop plants with desired traits for agronomic purposes with significantly less undesirable side effects on the plant genome. While traditional plant breeding methods involve chemical and radiation mutagenesis that often create random deleterious and chimeric mutations across genomes, modern gene‐editing tools allow precise modification of the genome at a desired position (Lowder *et al*., 2015; Malzahn *et al*., 2017; Xiong *et al*., 2015). Genome modification requires an engineered nuclease to create double‐strand breaks (DSBs) at defined targets, which then triggers cellular DNA repair mechanism, depending on the DNA repair pathway and presence of a repair template. There are two known DSB repair pathways, nonhomologous end joining (NHEJ) and homologous recombination (HR). NHEJ in most instances leads to random insertions or deletions (indels) of nucleotides at the repair site. In case DSB generates overhangs, NHEJ can also introduce gene insertions or precise gene modifications with a double‐stranded DNA fragment with compatible overhangs (Cristea *et al*., 2013; Maresca *et al*., 2013). In the presence of a DNA template with homology to the separated chromosome ends, DSBs can be repaired by HR, although this mechanism is rather exceptional at least in somatic cells. Nevertheless, this process can be used to insert DNA fragments and precisely modify genes (Bortesi and Fischer, 2015).

Zinc finger nucleases (ZFNs) and transcription activator‐like effector nucleases (TALENs) have shown promising results in achieving site‐directed DNA breaks. Both enzymes use a dimeric *Fok*1 nuclease for creating DNA breaks (Christian *et al*., 2010; Smith *et al*., 2000). In 2013, the type II *clustered regularly interspaced short palindromic repeat* (CRISPR)‐associated Cas9 system was discovered in *Streptococcus pyogenes* (*Sp*), which emerged as a powerful tool to induce precise mutations in the human genome (Cong *et al*., 2013; Mali *et al*., 2013). It promises high on‐target activity and low off‐target effects compared to RNAi (Smith *et al*., 2017). Subsequent implementation of *Sp*Cas9 as RNA‐guided, sequence‐specific nuclease (SSN) for genome editing in plants led to comparably fast and reliable results (Li *et al*., 2013). *Sp*Cas9‐mediated DNA editing involves introduction of two components, the Cas9 protein and a synthetic single guide RNA (sgRNA), into the target cell (genome) to be mutated. The sgRNA (~80 nucleotide [nt] total length) consists of a ~20 nt sequence with sequence similarity to the target gene and a synthetic RNA sequence that adopts functions of CRISPR RNA (crRNA) and transactivating crRNA (tracrRNA) of the original bacterial system (Deltcheva *et al*., 2011; Jinek *et al*., 2012; Sorek *et al*., 2013). *Sp*Cas9 induces DSBs by recruiting the sgRNA. An important requirement for DNA cleavage is the presence of a conserved protospacer adjacent motif (PAM), usually carrying the sequence 5′‐NGG‐3′ (for *Sp*Cas9) downstream of the target DNA (Gasiunas *et al*., 2012; Jinek *et al*., 2012). Since 2013, the *Sp*Cas9 system has successfully been applied for gene‐editing in plants such as *Arabidopsis thaliana*, tobacco and tomato (Brooks *et al*., 2014; Li *et al*., 2013; Nekrasov *et al*., 2013), as well as cereals such as rice, wheat, barley and sorghum (Jiang *et al*., 2013; Miao *et al*., 2013; Zhang *et al*., 2014; reviewed in Ma *et al*., 2016; Malzahn *et al*., 2017). Engineering disease resistance in major crops is especially promising because many resistant traits are recessively inherited (Hückelhoven *et al*., 2003; van Schie and Takken, 2014). A prominent example is powdery mildew resistance in cereals, which is conferred by recessive alleles of the locus *mildew‐o* (*mlo*; Acevedo‐Garcia *et al*., 2014). Significantly, *Sp*Cas9‐mediated simultaneously editing each of the three *Mlo* homeologs in allohexaploid bread wheat (*Triticum aestivum*) resulted in *mlo*‐based disease resistance against the wheat powdery mildew fungus *Blumeria graminis* f. sp. *tritici* (Wang *et al*., 2014). A limitation of the technology was the low mutation frequencies shown in the above study for wheat (5.6% in the T0 generation). Plant RNA Pol III‐dependent promoters from small nuclear RNA (snRNA)‐encoding genes (e.g. *U3* snRNA and *U6* snRNA) have been used to express sgRNA that guides the Cas9 protein to its target in the genome (Brooks *et al*., 2014; Jiang *et al*., 2013; Li *et al*., 2013; Miao *et al*., 2013; Nekrasov *et al*., 2013; Zhang *et al*., 2014). Lawrenson *et al*. (2015) exploited the wheat promoter of the *TaU6* snRNA gene for *Sp*Cas9‐mediated gene‐editing of barley *HvPM19*, which encodes an ABA‐inducible plasma membrane protein. Holme *et al*. (2017) edited *HvPAPhy*, a barley phytase gene using a similar construct. Mutation frequencies of 10%–44% were observed in the T0 generation, and induced mutations were transmitted to T1 plants independently of the T‐DNA construct. Kapusi *et al*. (2017) used the *Sp*Cas9 system to disrupt a barley *Endo‐N‐acetyl‐*β*‐D‐glucosaminidase* (*ENGase*) gene by employing the rice *OsU6* promoter to drive the sgRNA, reaching a *Sp*Cas9‐induced mutation frequency of 78%. However, these studies on cereals had some limitations concerning the mutation and/or transformation efficiency, thereby either accessing a mutation enrichment method using restriction enzymes to identify mutated plants in T0 generation (Holme *et al*., 2017; Lawrenson *et al*., 2015) or studying a large number of explants (Kapusi *et al*., 2017) to identify *Sp*Cas9‐positive plants (~10%), which reduces the overall efficiency of the *Sp*Cas9 gene‐editing system. These results indicate a need to improve the efficiency of *Sp*Cas9‐mediated gene‐editing in cereals.

In this study, we exemplarily used *HvMORC1* (GenBank: HG316119.1), one of the seven members of the barley *microrchidia* (*MORC*) GHKL (gyrase, Hsp90, histidine kinase, MutL) ATPase subfamily (Koch *et al*., 2017), to further improve application of the *Sp*Cas9‐mediated gene‐editing system in the cereal model barley. Plant *MORC* genes were first discovered in a genetic screen for *Arabidopsis* knockout (KO) mutants with compromised resistance against the turnip crinkle virus (TCV), suggesting that they play a role in plant immunity (Kang *et al*., 2008, 2010, 2012). Subsequent studies in Arabidopsis revealed their involvement in gene silencing and transposable element repression (Lorković *et al*., 2012; Moissiard *et al*., 2012, 2014). Unlike Arabidopsis *atmorc* mutants, barley became more resistant to fungal pathogens, such as powdery mildew fungus *Blumeria graminis* f. sp. *hordei* (*Bgh*), when *HvMORC2*, a paralog of *HvMORC1*, was partially silenced by expressing *MORC2*‐targeting silencing constructs with inverted promoters in transgenic plants (Langen *et al*., 2014). Consistent with this, transient overexpression of either of the five at that time‐known *HvMORC* paralogs compromised resistance to *Bgh*. Yet, functional analysis of cereal MORC proteins has been hampered by the unavailability of respective KO mutants. Hence, we anticipated that the *MORC* gene family is an excellent model for *Sp*Cas9‐mediated gene‐editing applications in barley.

Using a novel barley *U3* snRNA promoter to drive the sgRNA, we achieved an unprecedentedly high mutation frequency. Distinct *hvmorc1*‐KO mutations were detectable by target sequencing in the transgenic calli, the T0 generation (77%) and T1 (70%–100%) generation, which represents an important improvement of the technology. Extending earlier findings that were based on *hvmorc2*‐KD mutants generated by RNAi‐mediated knockdown (KD) strategies, *Sp*Cas9‐edited *hvmorc1*‐KO barley showed increased disease resistance to biotrophic *Bgh* and necrotrophic *Fusarium graminearum*. However, in contrast to barley *hvmorc1*‐KD mutants, *hvmorc1*‐KO barley, alike *atmorc1* mutants, showed de‐repressed expression of transposable elements (TEs), suggesting that barley MORCs also are involved in genome stabilization.

## Results

### Identification and characterization of a barley RNA Pol III‐dependent snRNA promoter

**Figure 1:**
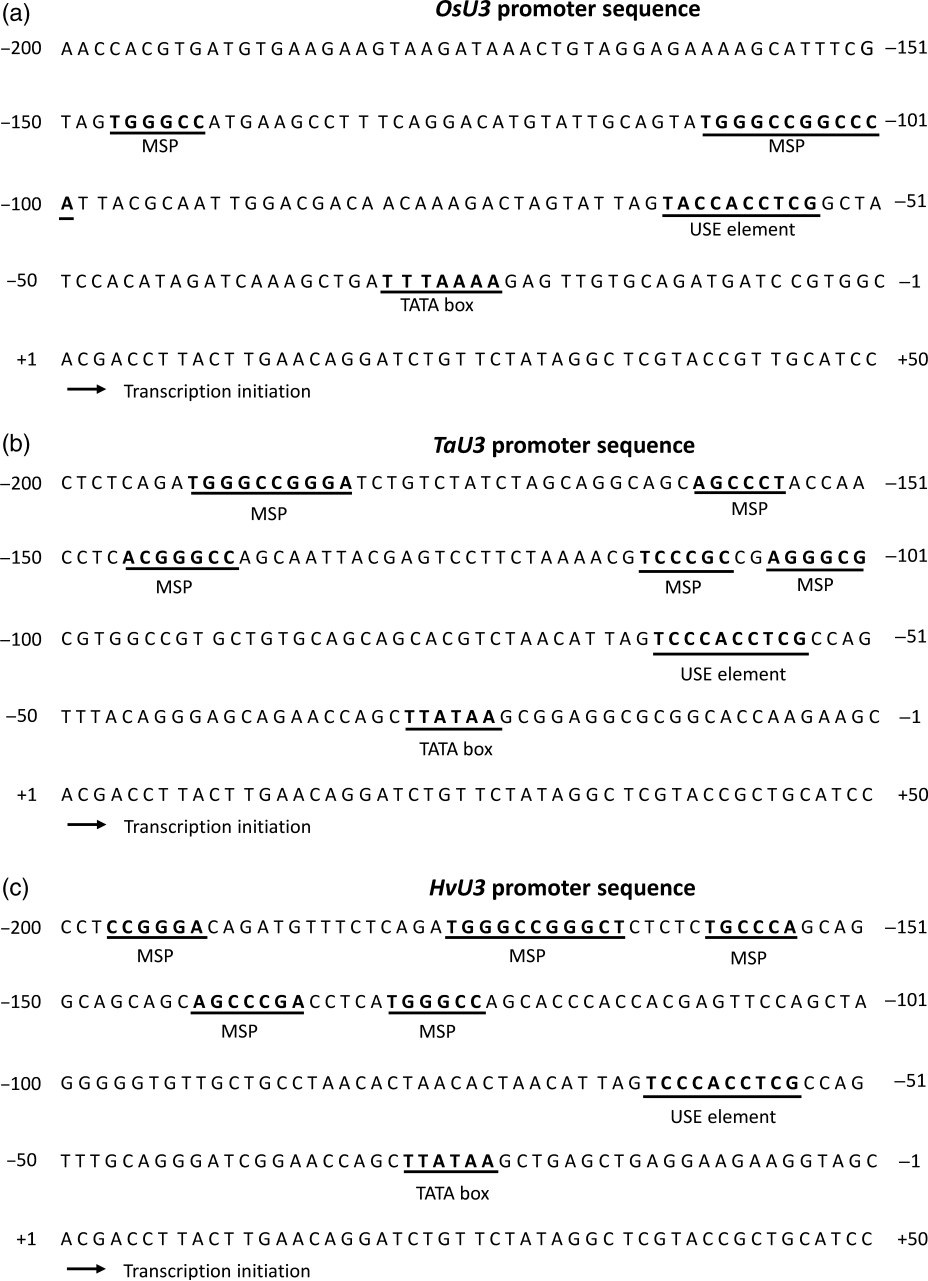
*U3* promoter sequences from rice (a); wheat (b) and barley (c). Sequence motifs are underlined: TATA box, upstream sequence element (USE) and monocot‐specific promoter (MSP) element.

As RNAi‐mediated KD may result in low efficiency and thus substantial residual amounts of transcript and protein, we further analysed the function of *MORCs* using stable KO mutant barley lines generated by *Sp*Cas9‐based nuclease. To ensure efficient transcription of sgRNAs in barley cells, we first set out to identify suitable regulatory elements by focusing on cereal *U3* snRNA promoters. A *U3* snRNA promoter from rice has been previously characterized (*OsU3*; Qu *et al*., 1996). This promoter has been used for *Sp*Cas9‐mediated gene‐editing in rice (e.g. Zhang *et al*., 2014) and maize (Xing *et al*., 2014). A suitable *HvU3* regulatory element (GenBank: CAJX011995286.1) was identified by similarity to the wheat *TaU3* promoter (GenBank: X63065.1; Marshallsay *et al*., 1992) from the database of barley cultivar ‘Bowman’ (http://webblast.ipk-gatersleben.de/barley/). *U3* snRNA promoter sequences from barley, rice and wheat were compared for the presence of features characteristic for Pol III‐dependent promoters in monocotyledonous plants: TATA box, an upstream sequence element (USE), and monocot‐specific promoter (MSP) elements (Figure 1a–c). MSPs are conserved G + C‐rich sequences with a consensus of RGCCCR in either direction, usually present −30 to −130 bp upstream of the USE (Connelly *et al*., 1994). Consistent with this, the barley *HvU3* promoter (p*HvU3*) contains a TATA box, located 23 base pairs (bp) upstream of the transcription initiation site, and the USE with the consensus sequence 5′‐TCCCACCTCG 25 bp upstream of TATA box, along with five MSPs, thus matching with the characteristics of RNA Pol III‐dependent promoters from monocotyledons (Waibel and Filipowicz, 1990).

### Assessment of barley and rice *U3* snRNA promoter activities

**Figure 2:**
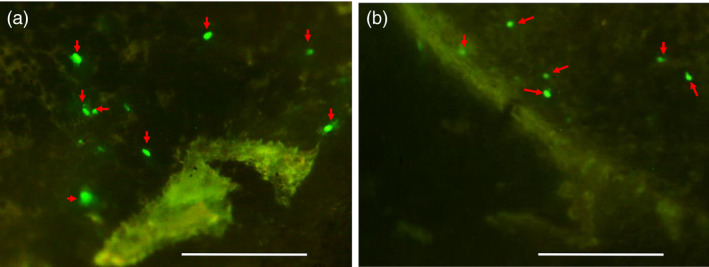
*GFP* expression in immature embryos of barley cv. Golden Promise 48 h after biolistic transformation with (a) pGY1‐p*H* *vU3*: *GFP*, containing 638 bp of the promoter sequence upstream of the coding region of the *U3* snRNA promoter from barley, and (b) pGY1‐p*O* *sU3*: *GFP*, containing 380 bp of the promoter sequence upstream of the coding region of the *U3* snRNA from rice, to drive *GFP* expression. Arrows mark GFP fluorescence. Bar scale 0.5 mm.

We first studied the activity of barley and rice *U3* snRNA promoter fragments in the tissue of immature barley embryos; 638 and 380 bp upstream of the predicted transcription start sites of *HvU3* and *OsU3*, respectively, were cloned into pGY1‐*35s*:*GFP* (Figure S1). The *U3* regulatory elements replaced the 35S promoter in pGY1 to drive expression of *GFP*. Resulting constructs were delivered to tissues from excised immature embryos of spring barley cultivar (cv.) Golden Promise by particle bombardment. Foci of GFP expression were detected 48 h after bombardment in embryonic cells transformed with either construct (Figure 2a,b). Although foci occurred at rather low frequencies in comparison with our routine observations, when bombarding constructs for Pol‐II promoter‐driven expression, the results demonstrate activity of both the *HvU3* and *OsU3* promoter fragments in barley. Notably, this also suggests that *U3*‐driven transcripts can, at least to some extent, engage the translational machinery in the cytoplasm.

### *Sp*Cas9‐induced mutation of *HvMORC1*

**Figure 3:**
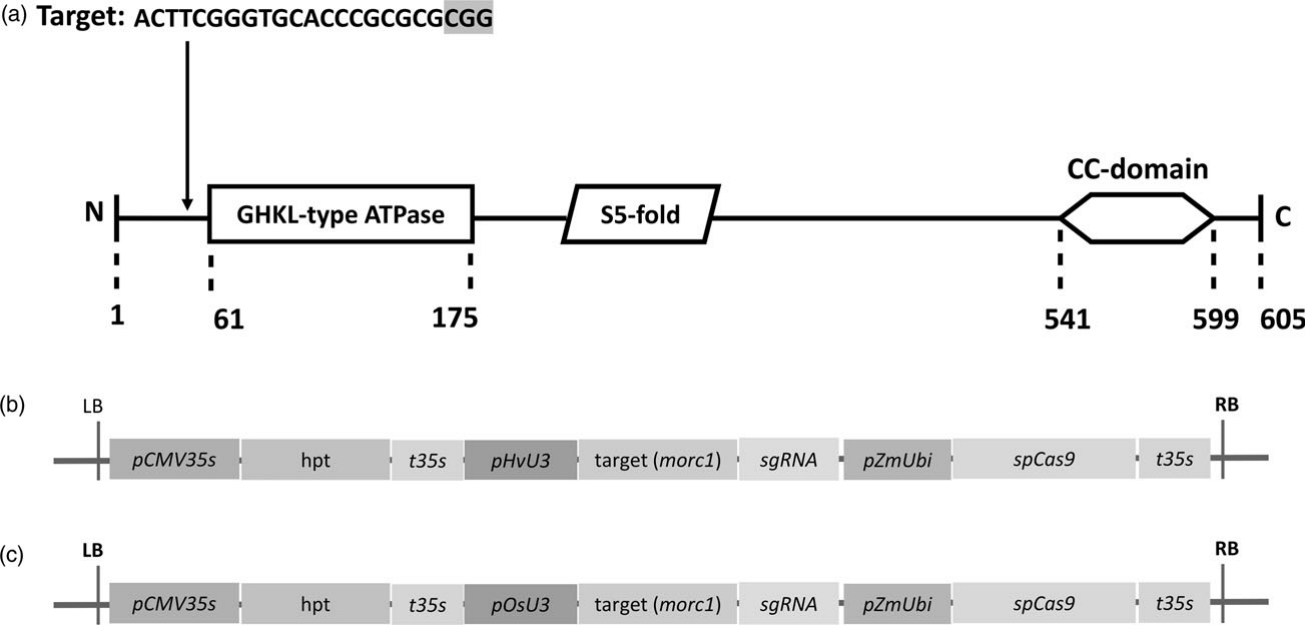
MORC domain structure and constructs used for targeted KO of *HvMORC1* by *Sp*Cas9‐mediated gene‐editing. (a) Targeted area (20 nt) of *HvMORC1* domain with the PAM sequence in grey shade. The hallmark domains of *HvMORC1*: a GHKL‐type ATPase, an S5‐fold and a CC‐domain, are highlighted. (b, c) Schematic representation of the T‐DNA regions containing all components for *Agrobacterium*‐mediated, *Sp*Cas9‐based *HvMORC1* gene‐editing. Construct with barley *U3* promoter (b) and construct with rice *U3* promoter (c). Hygromycin, *hygromycin phosphotransferase gene* [*hpt*]; pZmUbi, *ubiquitin* promoter of *Zea mays*; t35s, *CaMV* 35S terminator; LB, RB, left and right border sequences of the T‐DNA; sgRNA, synthetic single guide RNA.

**Figure 4:**
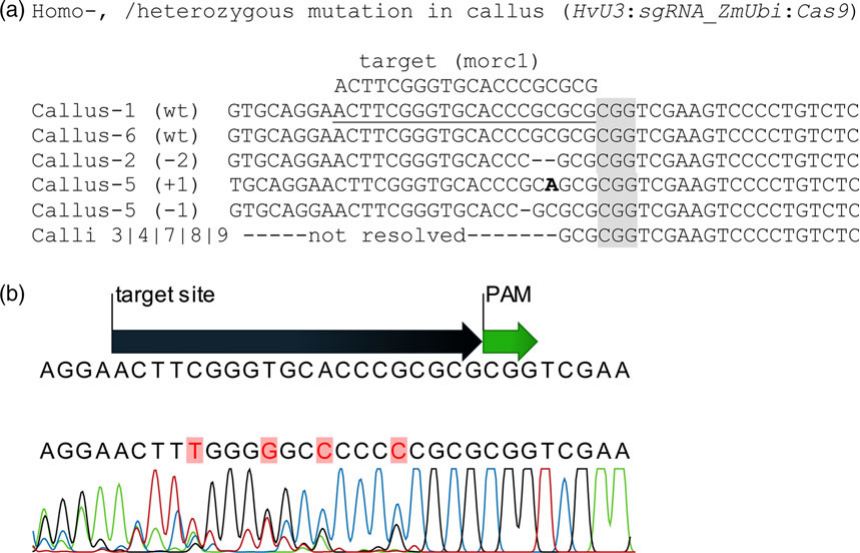
Mutations in the 20 bp target region of *HvMORC1* in 6‐week‐old T0 calli expressing the construct *HvU3:sgRNA* _*ZmUbi*:*Cas9*. (a) Overview of sequences obtained from calli. The PAM (CGG) sequence is highlighted in grey. (b) Example of a typical chromatogram, which could not be resolved into two or less distinct alleles. Note peaks with multiple overlaying signals indicating the presence of at least three different alleles within the sample.

The barley genome contains seven *MORC* genes, all of which are assumed to act as negative regulators of immunity as deduced from overexpression and RNAi‐mediated KD studies (Koch *et al*., 2017; Langen *et al*., 2014). To further address MORCs’ function in barley, *HvMORC1* was targeted by *Sp*Cas9 to generate loss‐of‐function alleles. A target site in the 5′ part of the *HvMORC1* gene upstream of the ATPase domain with no potential off‐targets in any of the seven barley *MORC*s (Figure S2) or the barley genome (see Experimental procedures) was chosen, and a respective sgRNA was designed (Figure 3a). Two constructs *HvU3:sgRNA* and *OsU3:sgRNA*, containing either p*HvU3* or p*OsU3* driving sgRNA expression and *Sp*Cas9 under control of the maize ubiquitin promoter (*ZmUbi*:*Cas9*), were transformed into immature embryos by agro‐transformation (Figure 3b,c; see also Figure S3). Genome editing activity from transformation of the construct with *HvU3*‐driven sgRNA was analysed in calli 6 weeks after transformation. Genomic DNA was extracted from randomly chosen embryonic calli grown on hygromycin selective media. The target region was amplified by PCR, and amplicons were analysed by direct sequencing of both strands. From two calli, the wild‐type (wt) sequence was obtained (Figure 4a; calli 1 and 6), and chromatograms were not indicative of any nuclease activity at the target site. In contrast, a homozygous deletion of 2 bp was obtained for one callus and also a bi‐allelic lesion (Figure 4a, calli 2 and 5). For the remaining five calli, varying degrees of nuclease activity were detected in chromatograms, and precise alleles could not be deduced as multiple sequences were detected (Figure 4a,b), suggesting that samples used for PCR originated from a mixed population of cells containing different molecular lesions at the target site. Thus, nuclease activity was detected in almost 77% (7/9) of analysed calli by direct sequencing, and bi‐allelic disruptive mutations affecting both alleles were also readily detected, suggesting occurrence of loss‐of‐function lines already in the T0 generation. We used TIDE (Tracking of Indels by Decomposition; Brinkman *et al*., 2014) to further access the spectrum and frequency of *Sp*Cas9‐induced mutations in calli. Overall, mainly indels of −2, −1 and +1 nt were detected by decomposition of chromatograms, and frequencies were comparable. Deletions of up to 5 nt were also detected, but frequencies were low. Among all calli (including those without detectable mutations), the wt sequence represented in average only 40% (±34%) of all sequence information, suggesting highly efficient genome editing when expressing sgRNAs under control of the *HvU3* promoter.

### Selection of homozygous *hvmorc1*‐KO barley in the T1 generation

**Figure 5:**
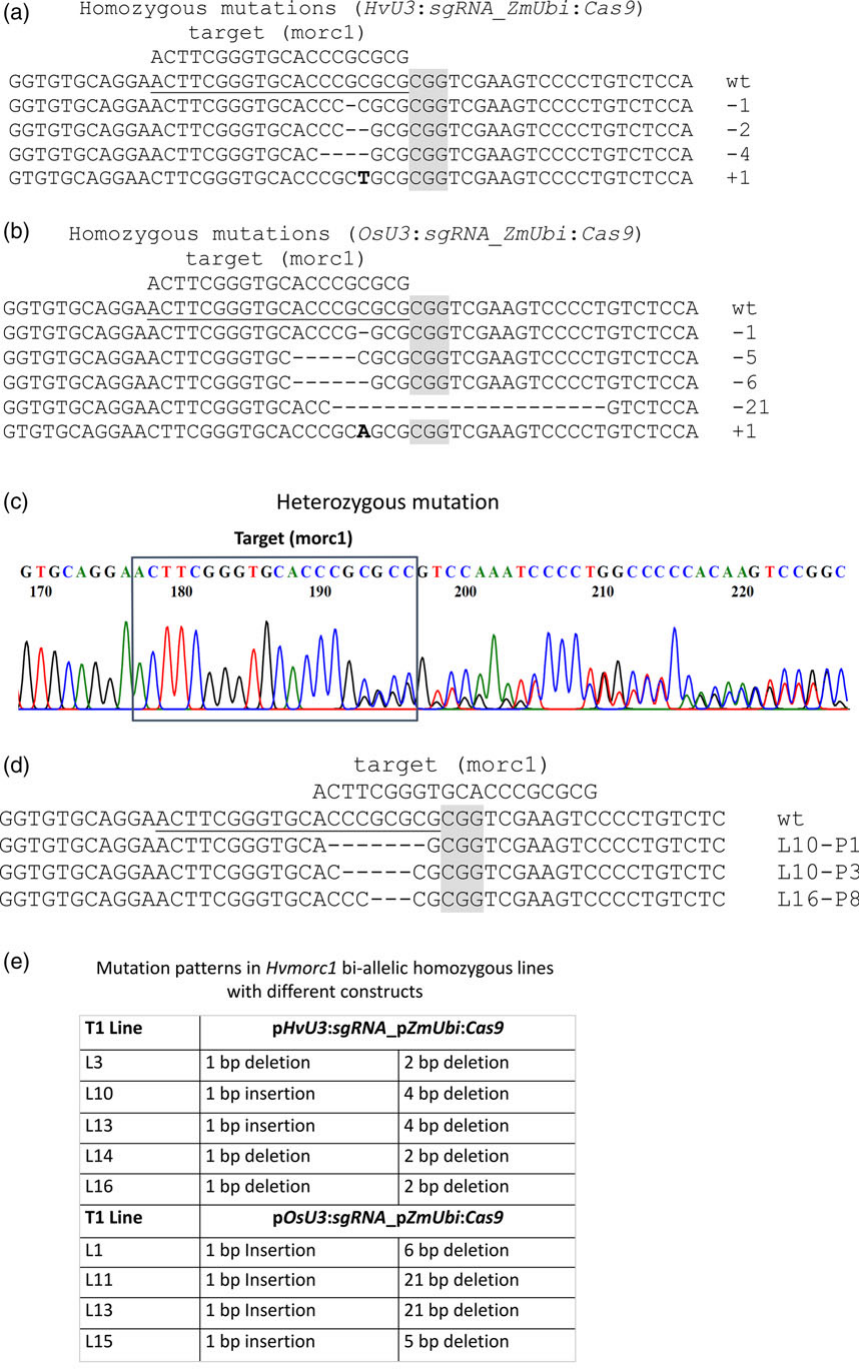
*Sp*Cas9‐induced mutations in independent barley T1 lines. (a–b) Homozygous mutations in T1 plants containing the *HvU3:sgRNA* _*ZmUbi*:*Cas9* and the *OsU3:sgRNA* _*ZmUbi*:*Cas9* construct, respectively. (c) Heterozygous mutants have characteristic double peaks in the chromatogram, for example starting 4 bp upstream of the PAM sequence (grey). (d) Mutation patterns of heterozygous mutants determined after sequencing using specific primers (Table S1) from both directions. (e) Examples for mutations in homozygous bi‐allelic T1 lines. The PAM (CGG) sequence is highlighted in grey, the 20 bp target region in *HvMORC1* is underlined, and insertions are marked in bold.

Genome editing activities were further analysed in T1. T0 plantlets regenerating on hygromycin selective medium were randomly selected and propagated in soil to obtain seeds for T1 generation. Notably, the parental T0 lines were not checked for the presence of either T‐DNA construct or mutations in *HvMORC1*. In T1 offspring, target sites were PCR‐amplified and amplicons were analysed by sequencing. For construct *HvU3*:sgRNA, 71 2‐week‐old plants from 12 different T1 lines (five to six plants per line) were analysed, and mutations could be detected in all T1 populations (100% efficiency). Similarly, 60 plants from 10 independent T1 lines (six plants per line) carrying *OsU3*:sgRNA were analysed. Mutations were detected in seven of these T1 populations (70% efficiency). For both transformation events, homozygous indel mutations (Figure 5a,b) were identified within the 20 bp target sequence, at a frequency of 38% (*HvU3*) or 42% (*OsU3*). Heterozygous mutations showed the presence of double peaks in the sequencing chromatogram (Figure 5c,d). While homozygous mutations are bi‐allelic, heterozygous mutations theoretically could be either mono‐allelic or bi‐allelic (with different mutations on both chromosomes). Further assessment of T1 populations with bi‐allelic mutations showed that in each population plants homozygous for each allele could be discovered (Figure 5e). These results confirm that *Sp*Cas9 can induce different mutations on different chromosomal strands of the same T0 plants, resulting in homozygous plants with two different mutation patterns in the T1 generation. Notably, we also identified mutated T1 plants that did not contain a T‐DNA construct (nontransgenic): 11 of 73 tested plants (15%) were devoid of the construct, indicating segregation of T‐DNA construct and the lesions within *HvMORC1*.

### Enhanced sgRNA accumulation by *HvU3 promoter*‐driven expression in barley

**Figure 6:**
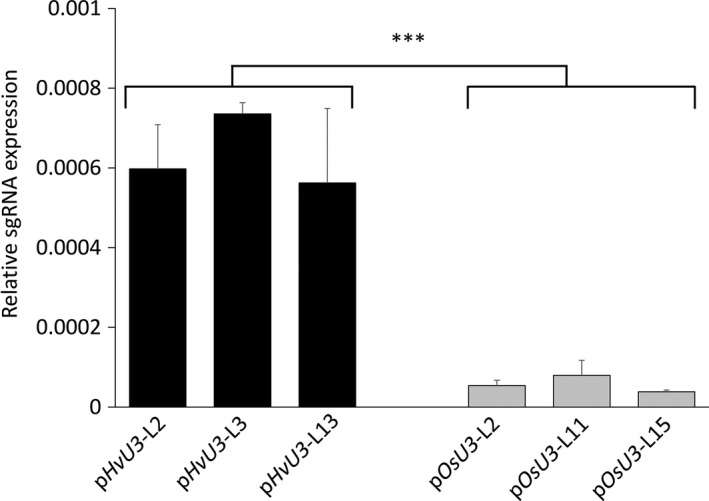
Relative expression of sgRNA under control of barley and rice *U3* promoters (p*H* *vU3* and p*O* *sU3*) in leaves of *hvmorc1*‐KO T2 homozygous mutants measured by RT‐PCR and normalized against *Sp*Cas9. Error bars indicate standard deviation of three repetitions. Asterisks indicate statistical significant difference (Student's *t*‐test: ***P < 0.001).

To further corroborate the suitability of the *HvU3* promoter for genome editing approaches in barley, expression of sgRNA under control of either the *HvU3* or *OsU3* promoter was quantified in T‐DNA‐positive lines using quantitative RT‐PCR (RT‐qPCR). To normalize for potential copy number variations and/or transgene insertions at different genome locations, sgRNA expression was normalized to the expression of T‐DNA‐encoded *Sp*Cas9 (Figure 6) or *hygromycin phosphotransferase* (Figure S4). We observed clearly higher expression of p*HvU3*‐driven sgRNA transcripts compared to p*OsU3* under both instances.

### *hvmorc1*‐KO mutants show increased resistance to fungal pathogens

**Figure 7:**
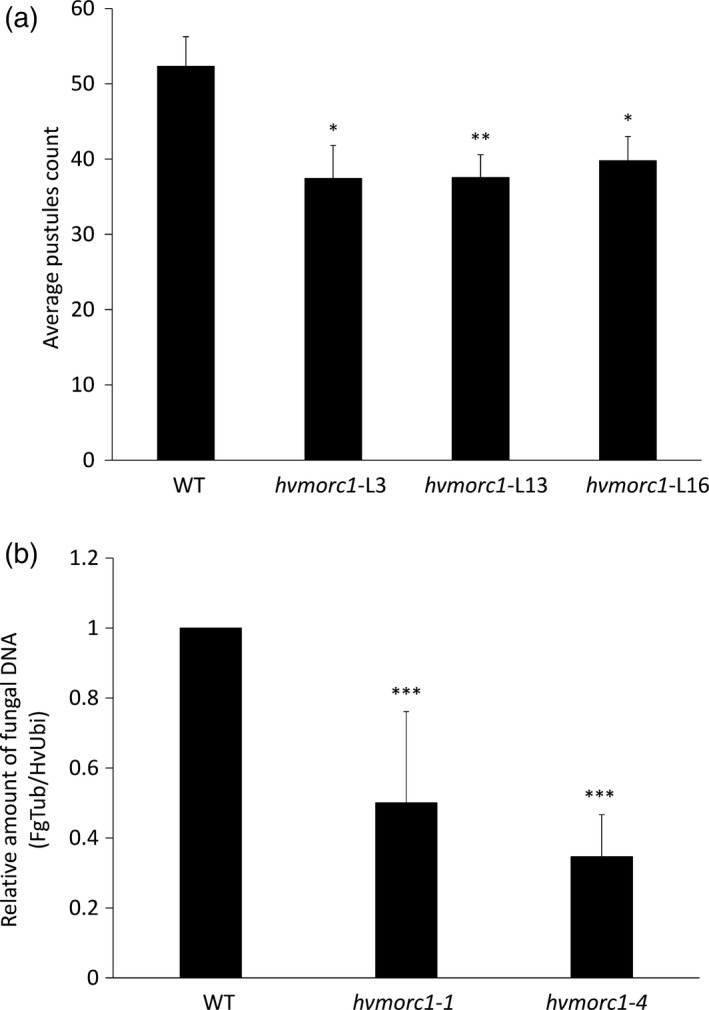
*Sp*Cas9‐mediated KO of *HvMORC1* results in enhanced resistance against fungal pathogens. (a) *hvmorc1*‐KO T1 barley cv. Golden Promise lines (*hvmorc1‐*L3, *hvmorc1*‐L13, *hvmorc1*‐L16) display enhanced resistance against powdery mildew. Detached second leaves of 14‐day‐old plants were inoculated with 3–5 conidia per mm^2^. *Bgh* colonies were counted at six dpi. Shown is the average number of *Bgh* colonies on 1.5 cm^2^ leaf area (*n* = 14). The experiment was repeated twice with similar results. Error bars indicate standard error. Asterisks indicate statistical significant difference (Student's *t*‐test *P < 0.05, **P < 0.01). (b) *hvmorc1*‐KO T2 homozygous mutants show enhanced resistance against *Fusarium graminearum* (*Fg*). For inoculation, 20 ul of *Fg* conidia (5 × 10^4^ conidia mL^−1^) was drop‐inoculated on detached third leaves of 21‐day‐old plants. Quantification of *Fg* on leaves was performed five dpi by quantitative RT‐PCR based on the ratio of fungal tubulin (*FgTub*) to plant ubiquitin (*HvUbi*). Significant changes are marked: ***P < 0.001 (Student's *t*‐test). Presented are mean of 10 leaves. Bars represent standard deviation of three repetitions.

Arabidopsis lines deficient in *MORC1* and *MORC2* are severely impaired in resistance to viral, bacterial, oomycete and fungal pathogens (Kang *et al*., 2008, 2012), while, in contrast, RNAi‐mediated reduced transcript levels of *HvMORC2* in barley enhanced resistance (Langen *et al*., 2014). To substantiate the opposing function of MORCs in barley vs. Arabidopsis, mutated *hvmorc1*‐KO T1 plants (consisting of both homozygous and heterozygous bi‐allelic mutations) from p*HvU3*:*sgRNA*_p*ZmUbi*:*Cas9* construct (*hvmorc1‐*L3, *hvmorc1*‐L13 and *hvmorc1*‐L16; see Figure 5e) were tested for powdery mildew resistance. Detached leaves were inoculated with conidia of *Bgh*A6 (virulent on cv. Golden Promise). Mutant lines developed less fungal colonies 6 days postinoculation (dpi) compared to wt plants (*hvmorc1*‐L3: 71.5%; ‐L13: 71.8%; *hvmorc1*‐L16: 76%; Figure 7a). These results were consistent with our expectation that barley MORC paralogs respond similar to *Bgh* (Langen *et al*., 2014). T1 plants from *hvmorc1*‐L10 and *hvmorc1*‐L13 that were homozygous for frameshift mutations (*hvmorc1‐1* and *hvmorc1‐4*; Figure S5) in the 5′ region of *HvMORC1* were propagated for analyses of T2 plants. We further studied *hvmorc1‐1* and *hvmorc1‐4* T2 homozygous plants for their response to the mycotoxin‐producing fungus *Fusarium graminearum*. Detached leaves of *hvmorc1*‐KO and wt plants were inoculated with macrospores of *F. graminearum*, and fungal DNA was quantified by quantitative PCR at five dpi. Fungal biomass was significantly reduced in *hvmorc1* KO mutant tissues (Figure 7b).

### *hvmorc1‐*KO mutants show enhanced expression of *PR* genes

**Figure 8:**
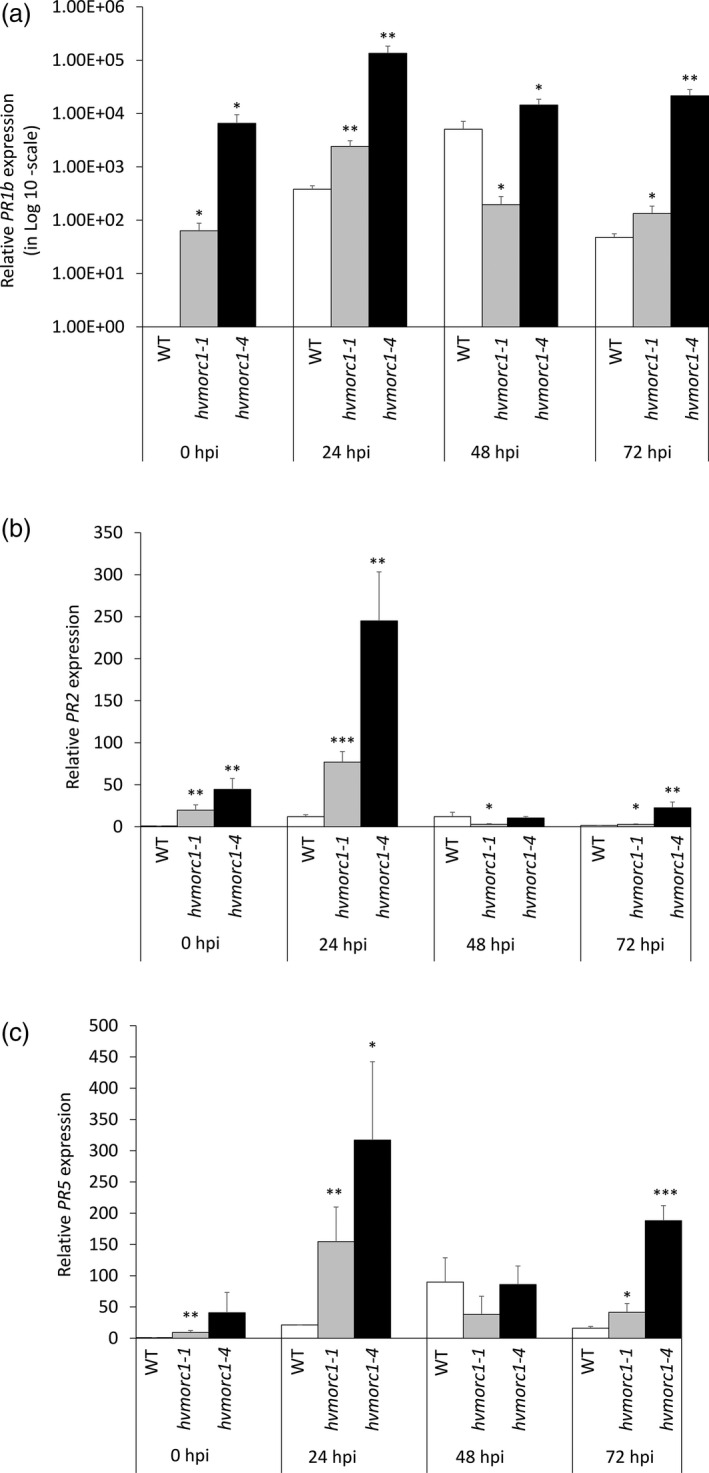
Relative *PR* gene expression in leaves of *Sp*Cas9‐generated *hvmorc1*‐KO T2 homozygous mutants vs. wt measured by RT‐qPCR and normalized to plant *ubiquitin*. Expression of SA pathway marker genes *HvPR1b* (a), *HvPR2* (b) and *HvPR5* (c). Detached second leaves of 14‐day‐old plants were inoculated with 10 to 15 conidia per mm^2^ (*n* = 5). Error bars indicate standard deviation of three repetitions. Asterisks indicate statistical significant difference (Student's *t*‐test: *P < 0.05, **P < 0.01, ***P < 0.001).

We investigated whether enhanced resistance of *hvmorc1‐*KO lines are associated with constitutive activation of defence responses. To this end, we measured expression of defence‐related genes. Expression of *HvPR1b* (GenBank: X74940.1), *HvPR2* (GenBank: AF479647.2) and *HvPR5* (GenBank: AM403331.1) in *hvmorc1‐1* and *hvmorc1‐4* T2 homozygous plants was determined by RT‐qPCR at 0, 24, 48 and 72 hpi with *Bgh*. Without pathogen stimulus (0 hpi), all of these genes were expressed to higher levels in *hvmorc1‐*KO mutants compared to wt (Figure 8a–c). Upon *Bgh* inoculation, differences in *PR* expression of *hvmorc1*‐KO vs. wt were even more pronounced, noticeably at an early infection stage (24 hpi). Most strikingly, expression of *PR1b* was strongly induced in the *hvmorc1*‐KO mutant. We concluded that compromised *HvMORC1* functions de‐repress at least parts of the plant defence system.

### *hvmorc1‐*KO mutants show de‐repressed transposable elements

**Figure 9:**
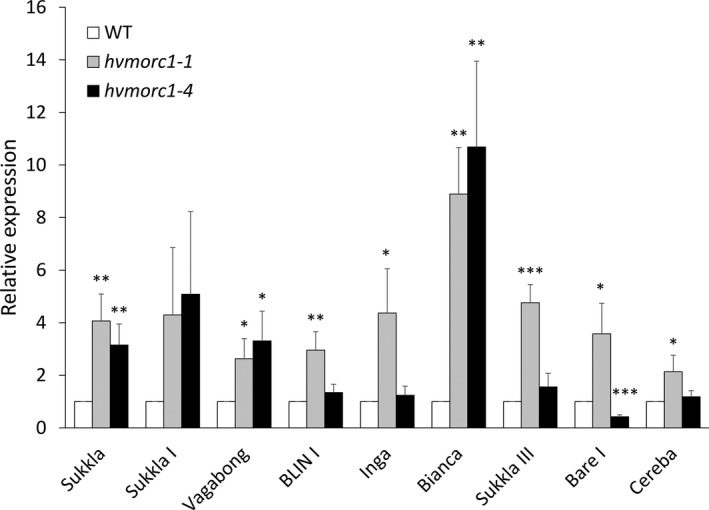
Expression of transposons (TEs) in second leaves of 14‐day‐old *Sp*Cas9‐generated *hvmorc1*‐KO T2 homozygous mutants (*hvmorc1‐1* and *hvmorc1‐4*) vs. wt assayed by RT‐qPCR and normalized to plant *ubiquitin* (*n* = 5). Error bars indicate standard deviation of three repetitions. Asterisks indicate statistical significant difference (Student's *t*‐test: *P < 0.05, **P < 0.01, ***P < 0.001).

In Arabidopsis *atmorc1* and *atmorc6* mutants, expression of transposable elements (TEs) located around the pericentromeric region is strongly increased (Moissiard *et al*., 2012), while transposon de‐repression has not been observed in barley *hvmorc1*‐KD mutants that were only partially silenced for *HvMORC1* (Langen *et al*., 2014). We refined the analysis of TE expression using the *hvmorc1‐*KO lines. To this end, expression of long terminal repeat (LTR) and non‐LTR retrotransposons (Long INterspersed Elements; LINE) with sequence similarity to those de‐repressed in Arabidopsis *atmorc* mutants (Langen *et al*., 2014; Moissiard *et al*., 2012) was measured by RT‐qPCR in *hvmorc1‐1* and *hvmorc1‐4* T2 homozygous mutated plants. In contrast to partially silenced *hvmorc1‐*KD mutants, *Sp*Cas9‐generated *hvmorc1‐*KO lines showed significant transposon de‐repression as compared to wt (Figure 9), although the degree of TE de‐repression was lower than previously reported for Arabidopsis *atmorc* mutants. This suggests that HvMORC1, such as AtMORC1, is involved in genome stabilization.

### *hvmorc1‐*KO mutants show increased expression of *HvMORC2* in immature embryos

**Figure 10:**
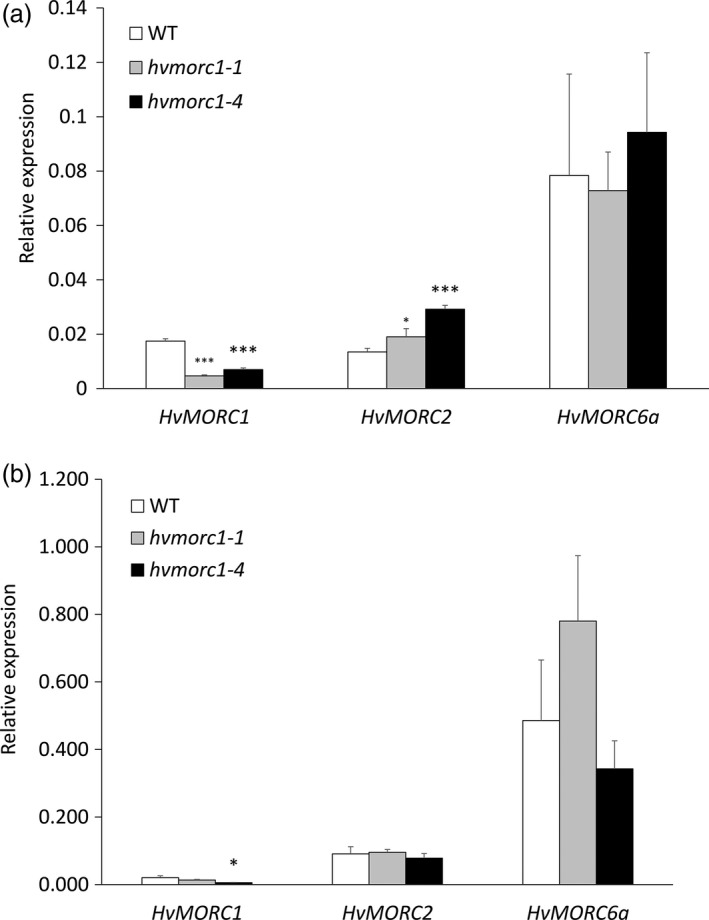
Relative expression of barley *MORC* genes in immature embryos and leaves of *hvmorc1‐* KO mutants and wt assayed by RT‐qPCR and normalized to plant *ubiquitin*. (a) Expression of *HvMORC1*, *HvMORC2* and *HvMORC6a* in immature embryos of T1 homozygous mutants (*hvmorc1‐1* and *hvmorc1‐4*). (b) Expression of *HvMORC1*, *HvMORC2* and *HvMORC6a* in leaves of T2 homozygous mutants (*hvmorc1‐1* and *hvmorc1‐4*). mRNA was extracted from second leaves of 14‐day‐old plants and immature embryos (*n* = 5). Error bars indicate standard deviation of three repetitions. Asterisks indicate statistical significant difference (Student's *t*‐test: *P < 0.05, ***P < 0.001).

In Arabidopsis, AtMORC1 and AtMORC2 interact with AtMORC6 to form distinct heteromers to achieve gene silencing. Additionally, the function of AtMORC6 is epistatic to both AtMORC1 and AtMORC2 (Moissiard *et al*., 2014). We assessed the effect of knocking out *HvMORC1* on the expression of other barley MORC homologs. Expression of *HvMORC2* (GenBank: HG316120) and *HvMORC6a* (GenBank: HG316122) was measured in immature embryos of T1 *hvmorc1*‐KO homozygous plants *hvmorc1‐1* and *hvmorc1‐4* and leaves of their T2 progenies. An increased expression of *HvMORC2* was observed in embryos of *hvmorc1‐1* and *hvmorc1‐4* compared to wt, while expression of *HvMORC6a* was similar in all genotypes (Figure 10a). This raises the possibility that, in the absence of HvMORC1, there is an increased expression of *HvMORC2* to maintain the cellular concentration of heteromeric complexes involving HvMORC6 for transcriptional repression of TEs, especially in immature embryonic tissue. Notably, *HvMORC2* showed no significant increase in expression in leaves of *hvmorc1*‐KO mutants (Figure 10b). Both immature embryos and leaves of *hvmorc1*‐KO show reduced transcript level of *HvMORC1* (Figure 10a,b), which could be a result of mRNA degradation by non‐sense‐mediated mRNA decay pathway that identifies and removes mRNA with premature STOP codons (Reviewed in Baker and Parker, 2004).

## Discussion

### Identification and transient expression of the barley *U3* snRNA promoter

Monocot and dicot RNA Pol III promoters from snRNA genes have been used to express sgRNA for genome editing. Diverse Arabidopsis promoters such as *AtU3b*, *AtU3d*, *AtU6*, *AtU6‐1*, *AtU6‐26* and *AtU6‐29* have been shown to be functional in dicotyledons (Brooks *et al*., 2014; Fauser *et al*., 2014; Feng *et al*., 2014; Gao *et al*., 2015; Ma *et al*., 2015; Mao *et al*., 2013; Nekrasov *et al*., 2013; Xing *et al*., 2014). For the expression of sgRNA in cereals, promoter variants *OsU3* and *OsU6* for rice (Ma *et al*., 2015; Shan *et al*., 2013), *TaU3* and *ZmU6* for maize (Svitashev *et al*., 2015; Xing *et al*., 2014), and *TaU6* for wheat and barley (Lawrenson *et al*., 2015; Wang *et al*., 2014) have been used. In the newly isolated barley *U3* promoter, the USE element lies 25 bp upstream of TATA box, which conforms to the consensus sequence of RNA Pol III‐dependent promoters (Figure 1c). In maize, deletion or substitution of MSPs decreases the transcription efficiency by 30%–60% (Connelly *et al*., 1994; Qu *et al*., 1996). Thus, it was crucial to check the functionality of promoters to be used in our *Sp*Cas9 system. RNA Pol III is able to produce functional mRNA with a low efficiency in human cells (Gunnery and Mathews, 1995), but similar studies have not been carried out in plants. In our study, upon transient transformation both barley and rice (RNA Pol III‐dependent) *U3* promoters coupled with *GFP* were expressed in barley immature embryos (Figure 2a,b), confirming their functionality. This finding also suggests that in plants some protein‐coding genes might be RNA Pol III‐dependent.

### Highly efficient genome editing in barley by *HvU3*‐driven sgRNA expression

Several studies have been published that reported the use of *Sp*Cas9 system in barley (Holme *et al*., 2017; Kapusi *et al*., 2017; Lawrenson *et al*., 2015). We used a single T‐DNA vector similar to the study by Lawrenson *et al*. (2015), main difference to this previous work being the use of the *U3* promoters of either barley or rice for sgRNA expression (Figures 1 and 3). Using these promoters, we achieved both stringent selection of transgenic plants on hygromycin and highly efficient genome editing with bi‐allelic mutations occurring already in T0 (Figure 4a). Although we cannot exclude an extraordinary high efficiency of the sgRNA used in our study, we assume that *U3* promoters are highly suitable for sgRNA expression in barley genome editing applications. Notably, *HvU3*‐driven sgRNA showed highest transcript accumulation as compared to *OsU3* (Figures 6 and S4). We provide these regulatory elements, and also *TaU6* and *OsU6* promoter fragments used in previous experiments, as part of a convenient toolkit to the plant research community. Our toolkit, which is similar to a previously reported toolkit for genome editing in dicot plants (Ordon *et al*., 2017), provides simple and rapid (Golden Gate‐based) cloning procedures and high multiplexing capacity for expression of four or up to eight sgRNAs. A description of the toolkit with cloning manual (Appendix S1) and vector maps (Appendix S2) is provided.

### *Sp*Cas9 nuclease‐induced mutations in barley

The aim of our study was to further increase the mutation frequency and select mutated plants growing on hygromycin selective medium using simple PCR and Sanger sequencing. Using *HvU3*, we obtained 77.7% mutation frequency in 6‐week‐old T0 callus (Figure 4a), which is seemingly high for barley. In separate experiments, T0 plants growing on hygromycin selective medium were selected for seed propagation. Later in T1 offspring of those plants, we obtained mutation frequencies of 100% and 77% in *HvMORC1* using *HvU3* and *OsU3* promoters, respectively. We obtained 38%–41% bi‐allelic homozygous plants and 15% plants were T‐DNA‐free T1 generation. The T‐DNA‐free homozygous plants do not contain any inserted DNA fragment/gene and carry the same mutation on both chromosomes, thus being ideal for gene function studies. Hence, we show here that it is possible to get a high frequency of mutation in barley using the *Sp*Cas9 technique. No doubt in future this technology would be the first choice of gene modification for plant pathologists, breeders and biochemists.

### Higher *HvMORC2* expression compensates for KO of *HvMORC1* in *hvmorc1‐*KO embryos

Previous work suggested that AtMORC1 and AtMORC2 do not interact with each other but both interact with AtMORC6, leading to the proposal that AtMORC6 mediates gene silencing by forming mutually exclusive heterodimers with either AtMORC1 or AtMORC2, or as a homodimer (Liu *et al*., 2014; Moissiard *et al*., 2014), and the function of AtMORC6 is epistatic to both AtMORC1 and AtMORC2 (Moissiard *et al*., 2014). Supportive of the former reports, we found an increased expression of *HvMORC2* in immature embryos of *hvmorc1* compared to wt, while expression of *HvMORC6a* was not changed (Figure 10a). These data suggest that in the absence of HvMORC1, there is an increased expression of *HvMORC2* to maintain the cellular concentration of heteromeric complexes involving HvMORC6 for transcriptional repression of TEs, although it is not resolved whether the targets of MORC1‐MORC6a and MORC2‐MORC6a complexes are identical, different or overlapping. Notably, *HvMORC2* showed no significant increase in expression in leaves of *hvmorc1*‐KO (Figure 10b), suggesting that the cell machinery is epigenetically programmed to identify and compensate for defects in DNA methylation during reproductive stage. Finally, constitutive *HvMORC6a* expression is higher compared to *HvMORC1* and *HvMORC2* (Figure 10a,b), further arguing for a prominent cellular requirement of HvMORC6a.

### Barley MORC1 modulates plant immunity and regulates TE expression

While in Arabidopsis and potato, *MORC*s positively regulate resistance to microbial pathogens, they are negative regulators in tobacco and tomato (Kang *et al*., 2008, 2010, 2012; Manosalva *et al*., 2015). In barley, RNAi‐mediated KD of *HvMORC2* also resulted in higher resistance, resembling the situation in tobacco and tomato (Langen *et al*., 2014). Given MORC proteins are also influencing gene silencing, there remains a technical uncertainty to assess the loss of function using RNAi. In the present study, a complete KO of HvMORC1 also enhances plant immunity against fungal pathogens *Bgh* and *F. graminearum* (Figure 7a,b), confirming similar immune functions of barley paralogs MORC1 and MORC2. Enhanced resistance to fungal pathogens correlated with elevated transcript level of *PR* genes in *hvmorc1‐*KO mutants (Figure 8a–c). *PR* expression was further enhanced in response to *Bgh*, particularly during initial phase of fungal colonization, providing *hvmorc1*‐KO mutants an early advantage over wt plants. It appears *HvMORC1* controls at least part of the plant's immune system, possibly thereby avoiding autoimmune reactions of an overshooting defence system.

Several lines of evidence suggest that MORC proteins also have nuclear targets. For example, *in vitro* assays demonstrated that AtMORC1 and HvMORC1 bind DNA/RNA, display endonuclease activity and are transferred from cytoplasmic locations to the nucleus in response to PAMP signals such as flagellin (Kang *et al*., 2012; Langen *et al*., 2014). Furthermore, MORC proteins from a range of prokaryotes and eukaryotes have been shown to play roles in chromatin modification and/or DNA recombination and repair (Iyer *et al*., 2008; Pastor *et al*., 2014; Perry and Zhao, 2003). The identification of AtMORC1 and/or AtMORC6 in three independent forward genetic screens of Arabidopsis mutants defective for transcriptional gene silencing (TGS) provided the first insight into nuclear MORC protein function (Brabbs *et al*., 2013; Lorković *et al*., 2012; Moissiard *et al*., 2012). In plants, TGS plays an important role in repressing TEs, intergenic regions, DNA repeats and some genes; it is mediated by the RNA‐directed DNA methylation (RdDM) pathway (Law and Jacobsen, 2010; Matzke *et al*., 2009, 2015). RdDM utilizes small RNAs to recruit the DNA methylation machinery to targeted sequences. DNA methylation in turn leads to recruitment of histone‐modifying enzymes, and the combined effect of these repressive epigenetic marks establishes chromatin in a silenced state. De‐repression of silenced reporter genes as well as TEs was observed in most *atmorc* mutants, suggesting that these proteins play a role in epigenetic gene silencing (Bordiya *et al*., 2016; Brabbs *et al*., 2013; Harris *et al*., 2016; Lorković *et al*., 2012; Moissiard *et al*., 2012, 2014). In the present study, we found an increased expression of barley TEs in homozygous *hvmorc1*‐KO mutants (Figure 9). A huge part (84%) of the barley genome consists of mobile and repeat structures, 76% of which are retrotransposons. Some 99.6% of retrotransposons are long terminal repeat (LTR) transposons, while 0.31% are non‐LTR retrotransposons (International Barley Genome Sequencing Consortium (IBSC), 2012). Notably, RNAi‐mediated KD of *HvMORC1* did not result in detectable de‐repression of barley TEs (Langen *et al*., 2014), suggesting that the remaining MORC protein activity (degree of gene KD was approx. 50%) was sufficient to repress TEs, which can explain the different phenotypes of RNAi‐generated *hvmorc1*‐KD vs. *Sp*Cas9‐mediated *hvmorc1*‐KO plants. Yet, when comparing *morc1* mutants from barley and Arabidopsis, the different degrees of transposon de‐repression are conspicuous (barley up to 14‐fold [this study] vs. Arabidopsis up to 500‐fold [Moissiard *et al*., 2014;] as compared to the respective wt plants). However, in two subsequent studies, lower expression of transposons (*AtCopia28*/*RomaniaT5*) was observed in *atmorc1* (Moissiard *et al*., 2014; Zhang, 2016). Moreover, a previous report showed that barley retrotransposons are responsive to various biotic and abiotic environmental cues (Alzohairy *et al*., 2012). Consistent with our study, the barley LTRs did not show high transcript level in response to such triggers.

While the link between MORC proteins role in immunity and TGS is currently unknown, the discovery that *Pseudomonas syringae* pv. *tomato* (*Pst*) infection alters AtMORC1 binding at genomic regions preferentially associated with TEs provides an important clue (Bordiya *et al*., 2016). A growing number of studies suggest that TEs are key regulatory elements that control stress‐associated gene expression (Dowen *et al*., 2012). Thus, the finding that *Pst* infection reduces AtMORC1 binding at loci associated with heterochromatic TEs led Bordiya *et al*. (2016) to propose that loss of AtMORC1 binding at these sites disrupts a silencing complex and thus up‐regulates heterochromatic TE expression. The de‐repressed TEs could serve as enhancers of proximal gene expression in barley. It is tempting to speculate that elevated resistance of *hvmorc1*‐KO mutants results from barley MORC role in genome stabilization, which is attenuated in the mutants resulting in higher expression of TEs and concomitantly *PR* gene expression.

## Experimental procedures

### Plant material and fungal inoculation

Seeds of barley (*Hordeum vulgare*) cv. ‘Golden Promise’ were germinated for 3 days on filter paper. Seedlings were transferred to soil (Fruhstorfer Erde Typ T) and cultivated in a growth chamber at 22 °C/18 °C (day/night cycle) with 60% relative humidity and a photoperiod of 16 h (240 μmol/m^2^/s photon flux density). After complete emergence (12–14 day), the second leaves were detached, laid on 0.5% (w/v) water agar and inoculated with *Bgh*A6 (Langen *et al*., 2014) at a density of 2 to 5 conidia mm^−2^. For expression analysis, a high density of 10 to 15 conidia mm^−2^ was used. *F. graminearum* (strain 1003; Jansen *et al*., 2005) was regularly cultured on SNA (synthetic nutrient‐poor agar) plates containing 0.1% KH_2_PO_4_, 0.1% KNO_3_, 0.1% MgSO_4_·7H_2_O, 0.05% KCL, 0.02% glucose, 0.02% sucrose and 1.4% agar. Plates were incubated at room temperature under constant illumination from one near‐UV tube (Phillips TLD 36 W/08, http://www.philips.de) and one white light tube (Phillips TLD 36 W/830HF, http://www.philips.de). Sterile 0.02% Tween water (v/v) was poured on 2‐week‐old plates, and conidial suspension was scrubbed using a glass rod and filtered through a miracloth (Calbiochem, http://www.merck-chemicals.de). Conidia concentration was adjusted to 5 × 10^−4^ spore mL^−1^; 20 μL of spore suspension was drop‐inoculated on detached barley leaves kept on 0.5% water agar plates. Square Petri plates with detached leaves were kept at room temperature under one white tube (Phillips TLD 36 W/830HF, http://www.philips.de). Progression of infection was routinely monitored. For quantification of fungal invasion, leaf samples were harvested at 5 dpi and DNA was extracted (Doyle and Doyle, 1987), which was later used to determine the amount of fungal DNA by quantitative RT‐PCR.

### Generation of vectors to study barley and rice *U3* promoter activity using *GFP* reporter gene in transient assay system

A 638 bp upstream of barley *U3* coding sequence (GenBank: CAJX011995286.1) and a 380 bp sequence upstream of rice *U3* coding sequence (Miao *et al*., 2013) were amplified with primers containing restriction sites *XhoI* and *NcoI* (Table S1). Both barley and rice *U3* promoters were coupled with the reporter gene for the green fluorescent protein (*GFP*) by replacing the CMV35s promoter in pGY1‐*35s*:*GFP* (Schweizer *et al*., 1999) using restriction enzyme *XhoI* and *NcoI* to generate plasmid constructs—pGY1p*HvU3*:*GFP* and pGY1p*OsU3*:*GFP*.

### Generation of CRISPR/Cas9 constructs

Twenty bp target sequences with NGG (PAM) at 3′ end were selected using CRISPR sgRNA design online tool (https://atum.bio/eCommerce/cas9/input) for *HvMORC1* (GenBank: HG316119.1). The designed 20 bp target sequences was blasted (BlastN) against nucleotide collection of *Hordeum vulgare* (taxid: 4513) at NCBI for putative off‐targets, and ACTTCGGGTGCACCCGCGCG was selected. Cloning overhangs (*Hv*U3: agca/aaac; *Os*U3: ggca/aaac) were added and guide sequences cloned as hybridized oligonucleotides. To adapt *OsU3*, *HvU3*, *OsU6* and *TaU6* elements for the multiplexing system, existing *BsaI* and *BpiI* sites were removed, and promoter fragments were cloned together with a ccdB cassette and the sgRNA backbone into a pUC57 derivative as previously described (Ordon *et al*., 2017). Recipient vectors were assembled by modular cloning as previously described (Engler *et al*., 2014; Ordon *et al*., 2017). Details on cloning procedures and primer sequences are available upon request.

### Plant transformation

Plasmids were electroporated (Gene Pulser, Biometra) into *Agrobacterium tumefaciens* strain AGL1 (Lazo *et al*., 1991), and the resulting *Agrobacterium* was used to transform spring barley ‘Golden Promise’ as described (Imani *et al*., 2011; Tingay *et al*., 1997). Transient barley transformation was performed as described (Schweizer *et al*., 1999). Immature barley embryos were shot using a particle inflow gun (PDS‐1000/He, BIO‐RAD) with DNA‐coated on 1‐μm gold particles. One microgram of plasmid per shot was used with a rupture disc of 650 psi.

### DNA isolation and quantitative PCR analysis

DNA/RNA extraction and quantitative RT‐PCR were performed as described (Doyle and Doyle, 1987; Imani *et al*., 2011). Primer pairs used for expression analysis are listed in Table S1.

## Conflict of interest

The authors declare no conflict of interest.

## Supporting information


**Figure S1** Maps of constructed plasmid.
**Figure S2** Target site conservation in *MORC* genes.
**Figure S3** 638 bp sequence of barley RNA Pol III promoter (TATA box is underlined) (A); 380 bp sequence of rice RNA Pol III promoter (TATA box is underlined) (B); Sequence of sgRNA is underlined with terminator (C).
**Figure S4** Relative expression of sgRNA under control of barley and rice U3 promoter (p*HvU3* and p*OsU3*) in leaves of *hvmorc1*‐KO T2 homozygous mutants measured by RT‐PCR and normalized against Hygromycin gene.
**Figure S5** *Sp*Cas9‐induced frame‐shift mutations in *HvMORC1* leads to premature STOP codons Predicted *HvMORC*1 open reading frames (in red) with premature stop codons after Cas9 induced mutation (b‐d) compared to wt (A) using online tool (http://web.expasy.org/translate/).
**Table S1** Oligonucleotide primers used in this study (restriction sites are underlined).

**Appendix S1** Cloning manual for pMGE genome editing vectors.

**Appendix S2** Annotated sequence files (Genbank) for pMGE vectors.

Supplementary File
