# Supplementary material for: Further analysis of barley MORC1 using a highly efficient RNA‐guided Cas9 gene‐editing system
Source: Plant Biotechnol J. 2018 May 7;16(11):1892–903. doi: 10.1111/pbi.12924 (PMC6181210; doi:10.1111/pbi.12924)
Supplement: Supplementary file 1 — Figure S1 Maps of constructed plasmid. Figure S2 Target site conservation in MORC genes. Figure S3 638 bp sequence of barley RNA Pol III promoter (TATA box is underlined) (A); 380 bp sequence of rice RNA Pol III promoter (TATA box is underlined) (B); Sequence of sgRNA is underlined with terminator (C). Figure S4 Relative expression of sgRNA under control of barley and rice U3 promoter (pHvU3 and pOsU3) in leaves of hvmorc1‐KO T2 homozygous mutants measured by RT‐PCR and normalized against Hygromycin gene. Figure S5 SpCas9‐induced frame‐shift mutations in HvMORC1 leads to premature STOP codons Predicted HvMORC1 open reading frames (in red) with premature stop codons after Cas9 induced mutation (b‐d) compared to wt (A) using online tool (http://web.expasy.org/translate/). Table S1 Oligonucleotide primers used in this study (restriction sites are underlined). [file PBI-16-1892-s003.docx]

**
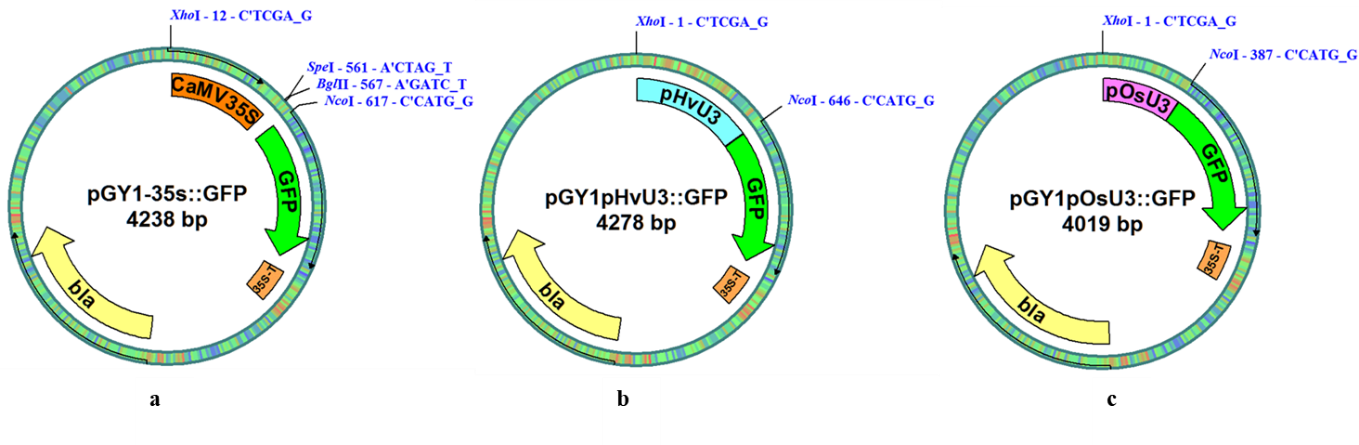
**

**Supplemental Figure S1.** Maps of constructed plasmid. (a) pGY1-*35s*:*GFP* plasmid used for promoter cloning; (b) Plasmid generated after coupling 638 bp of barley RNA Pol III promoter with *GFP*; (c) Plasmid generated after coupling 380 bp of rice RNA Pol III promoter with *GFP*.


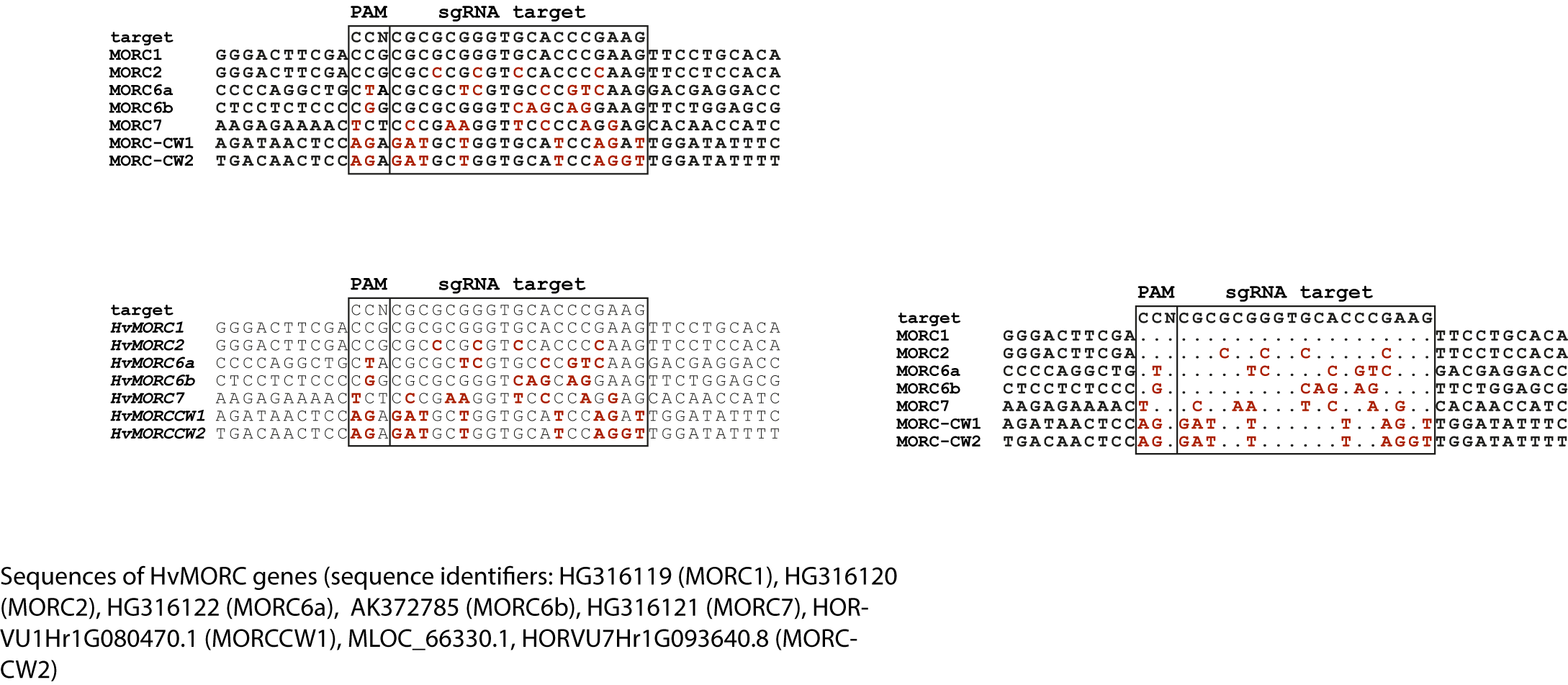


**Supplemental Figure S2.** Target site conservation in *MORC* genes. Alignment of potential target sites of the sgRNA in MORC family genes; mismatches to the sgRNA are displayed in red. Sequence identifiers: HG316119 (MORC1), HG316120 (MORC2), HG316122 (MORC6a), AK372785 (MORC6b), HG316121 (MORC7), HORVU1Hr1G080470.1 (MORCCW1), MLOC_66330.1, HORVU7Hr1G093640.8 (MORCCW2) (Koch et al. 2017, FiPS).


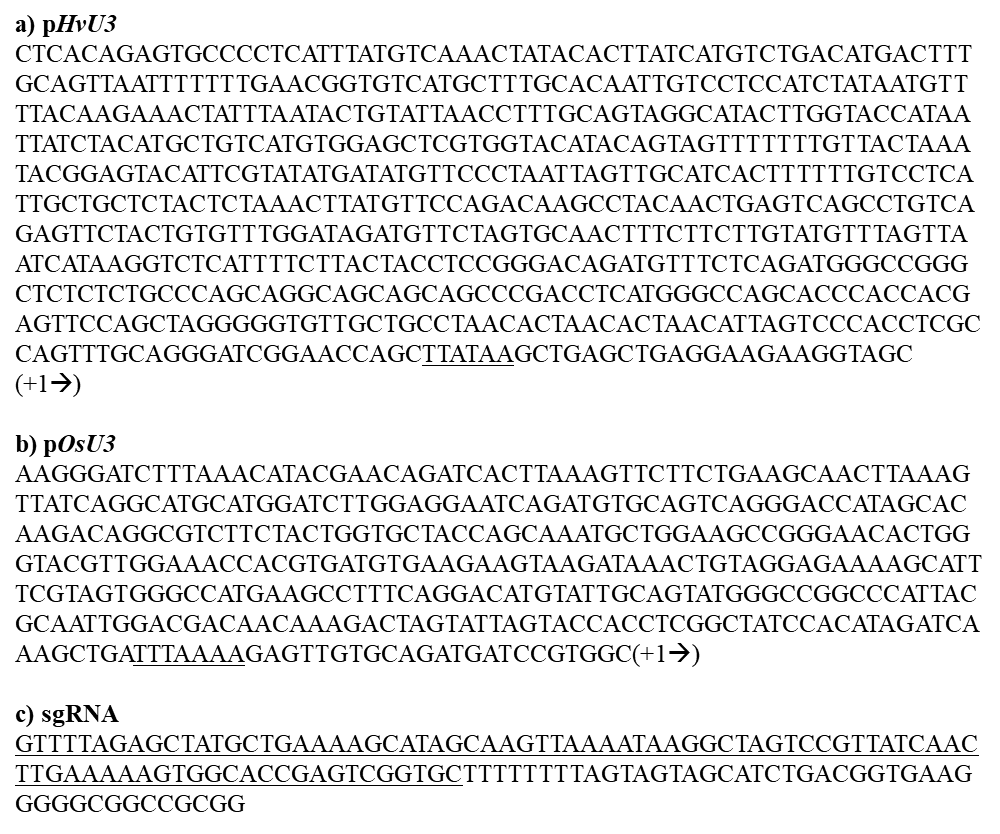


**Supplemental Figure S3.** 638 bp sequence of barley RNA Pol III promoter (TATA box is underlined) (A); 380 bp sequence of rice RNA Pol III promoter (TATA box is underlined) (B); Sequence of sgRNA is underlined with terminator (C).


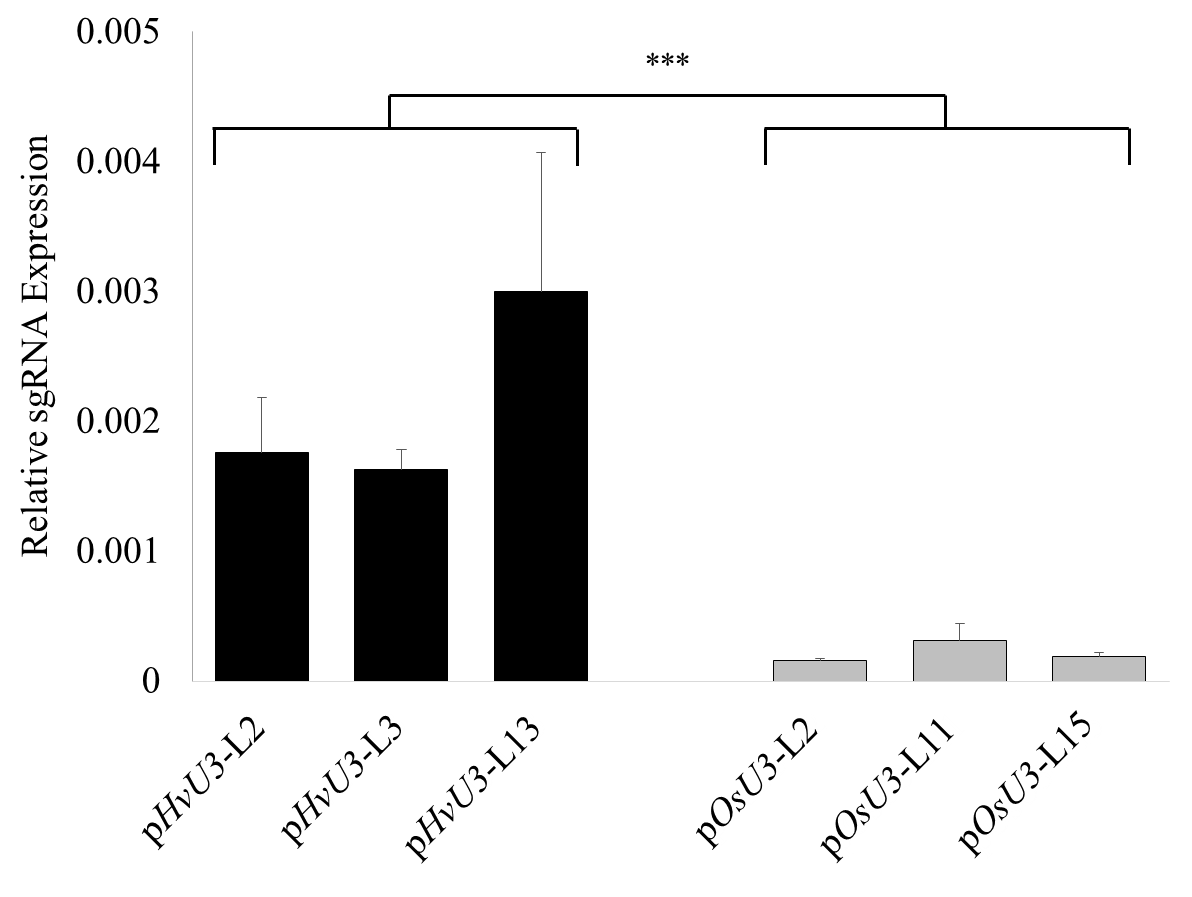


**Supplemental Figure S4.** Relative expression of sgRNA under control of barley and rice U3 promoter (p*HvU3* and p*OsU3*) in leaves of *hvmorc1*-KO T2 homozygous mutants measured by RT-PCR and normalized against Hygromycin gene. Error bars indicate standard deviation of three repetitions. Asterisks indicate statistical significant difference (Student’s t-test: *** P<0.001).

**
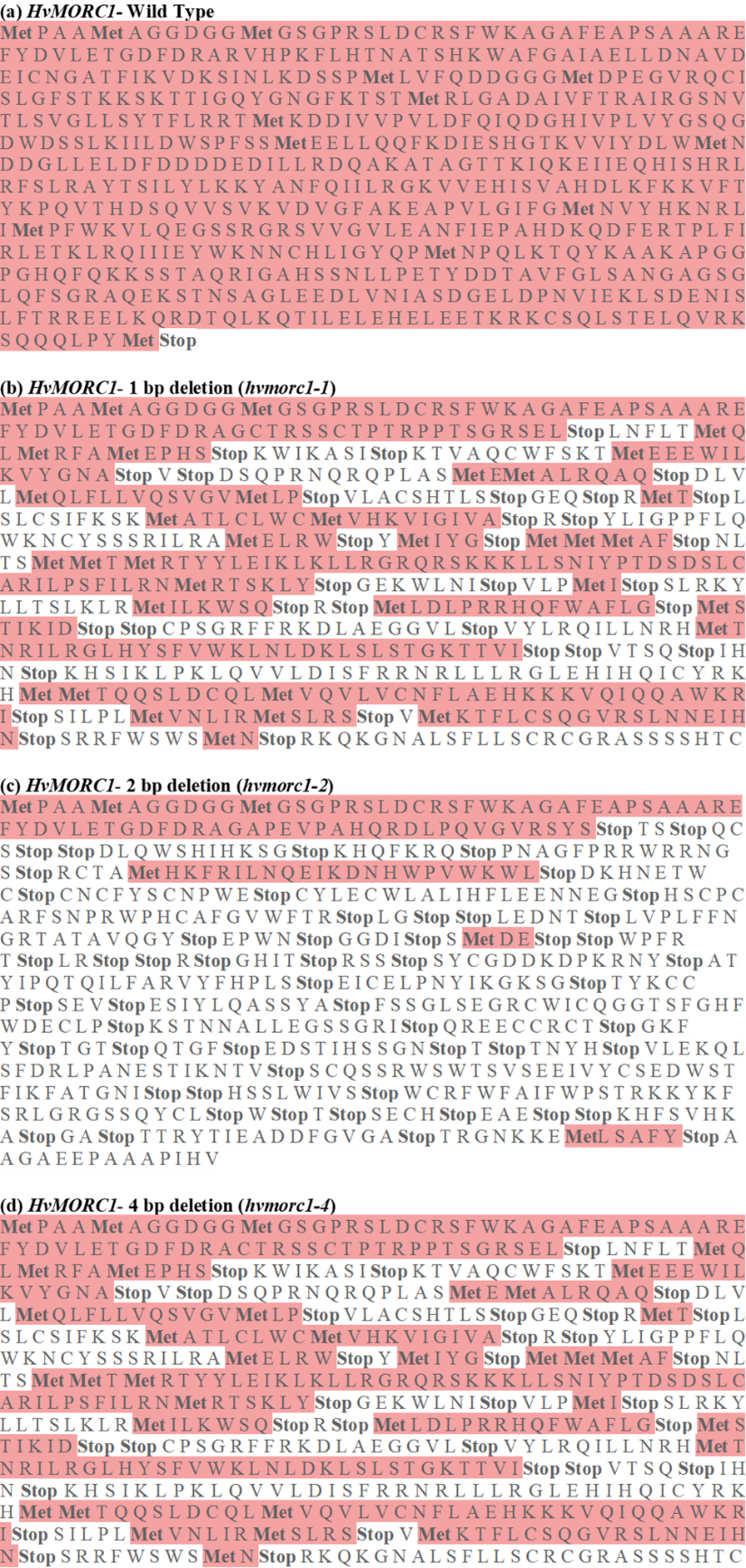
**

**Supplemental Figure S5.** *Sp*Cas9-induced frame-shift mutations in *HvMORC1* leads to premature STOP codons Predicted *HvMORC*1 open reading frames (in red) with premature stop codons after Cas9 induced mutation (b-d) compared to wt (a) using online tool (http://web.expasy.org/translate/).

**Table S1.** Oligonucleotide primers used in this study (restriction sites are underlined)

| Primer Name | Sequence 5’ -> 3’ | Target; Use |
| --- | --- | --- |
| JIHyg_F1 | GAACTCACCGCGACGTCTGTCGAG | Hygromycin resistance gene (*hpt*); T-DNA detection |
| JIHyg_R1 | CCGAACGCGCGTCTGCTGCTCCATA |  |
| Morc1S3F1 | GCGGCTCGCAGCCTTTTATT | Barley *MORC1;* Mutation detection |
| Morc1S3R1 | TTCCGATTACCTGACGCCCC |  |
| HvU3_Xho1_For | TATCTCGAGCTCACAGAGTGCCCCTCATT | Barley *U3* promoter; amplification |
| HvU3_Nco1_Rev | ATTCCATGGTGCTACCTTCTTCCTCAGCT |  |
| OsU3_Xho1_For | CGGCTCGAGAAGGGATCTTTAAACATACG | Rice *U3* promoter; amplification |
| OsU3_Nco1_Rev | ATTCCATGGGCCACGGATCATCTGCACAA |  |
| HvUbiquitin-F | TCGCCGACTACAACATCCAG | Barley ubiquitin; qPCR expression |
| HvUbiquitin-R | TGTGCTTGTGCTTTTGCTTC |  |
| FgTubulin-F | GGTCTCGACAGCAATGGTGTT | Fg tubulin; qPCR colonization |
| FgTubulin-R | GCTTGTGTTTTTCGTGGCAGT |  |
| Bianca-F | AAAGCGGGGAAATAAGCATT | TE; qPCR expression |
| Bianca-R | AGTCGTCGCATACCAATTCC |  |
| RLG_Vagabond-F | CCGTTCAATGGACCGATCAC | ’’ |
| RLG_Vagabond-R | ACGAGACTGAGCAACCTTCA |  |
| cereba-LP2 | GAAGATGAGCTTCCGTCGAG | ’’ |
| cereba-RP2 | GCGTGCACGAGCTCTAGTAA |  |
| BLIN-1F | TTCTGGGAGGACCGATGGAT | ’’ |
| BLIN-1R | TTGACTTGCGACGCTGTTTG |  |
| Bare1-F | ACGACACCTCCGCGTTCAG | ’’ |
| Bare1-R | CCGACCACATGCCTCCACGGTTTTTCCT |  |
| SUKKULA3-F | ACGACCAAGATGCGGTCCTTTCC | ’’ |
| SUKKULA3-R | AGACAGATGATCCCGACGGCAC |  |
| RLG_Sukkula-F | GGTTTTGGTGCGGTCTTGAT | ’’ |
| RLG_Sukkula-R | CTCCACGTCATCAAGGCATG |  |
| RLC-Inga-F | GCAAAGCCGCTCCTACTAAC | TE; qPCR expression |
| RLC-Inga-R | AACCCAGATAGCCCGTTCAA |  |
| SUKKULA1-F | TCTCAGAGTTGAGGTTTTCCAC | TE; qPCR expression |
| SUKKULA1-R | GTCAGACATAACCCCACCGTGTC |  |
| HvPR1b_F | GGACTACGACTACGGCTCCA | Barley *PR1b*; qPCR expression |
| HvPR1b_R | GGCTCGTAGTTGCAGGTGAT |  |
| HvPR2_F | TACTTCGCGTACCGTGACAA | Barley *PR*2; qPCR expression |
| HvPR2_R | GTGTAGGTCAGCCCGTTGTT |  |
| Hv2PR5_F | TAGAGCTTGCAGCAATGTCGACC | Barley *PR5a*; qPCR expression |
| Hv2PR5_R | CCTGAGCCCAGCTCGAAG |  |
| HvJMT_F | TGACTTCCCCAAAATGAAGG | Barley *JMT*; qPCR expression |
| HvJMT_R | CTTCCGAGAAACAGCTGAGG |  |
| HvMorc1_qPCR_F | GATGACATAGTTGTCCCTGTGC | *HvMORC1*; qPCR expression |
| HvMorc1_qPCR_R | GGCTCTCAATATCCTTGAACTGC |  |
| HvMorc2_qPCR_F | AGGTTCTGCTTTGCATTCGT | *HvMORC2*; qPCR expression |
| HvMorc2_qPCR_R | CTTCACGCCTTGTGAACAGA |  |
| HvMorc6a_qPCR_F | TGGAAACAGGCTGCGATGAT | *HvMORC6a;* qPCR expression |
| HvMorc6a_qPCR_R | CGCTACCAAAAGGGGACCAT |  |
| FgTubulin-F | GGTCTCGACAGCAATGGTGTT | *F. graminearum* tubulin; qPCR expression |
| FgTubulin-R | GCTTGTGTTTTTCGTGGCAGT |  |
| qPCRHyg_F | GGCGTCGGTTTCCACTAT | Hygromycin resistance gene (hpt); qPCR expression |
| qPCRHyg_R | GCTCCGCATTGGTCTTGA |  |
| qPCR*Sp*Cas9_F | GGCATTCTCCAGACCGTGAA | *Sp*Cas9; ; qPCR expression |
| qPCR*Sp*Cas9_R | TCGATCCTCTTCATGCGCTC |  |
| qPCRsgRNA_F | CGACTCGGTGCCACTTTT | sgRNA; qPCR expression |
| qPCRsgRNA_R | TGGGCGCGCGTTTTAGAG |  |

**References:**

Koch, A., Kang, H.-G., Steinbrenner, J., Dempsey, D.A., Klessig, D.F., Kogel, K.-H. (2017) MORC proteins: novel players in plant and animal health. *Front Plant Sci*. **8,** 1720.
