# Supplementary material for: Further analysis of barley MORC1 using a highly efficient RNA‐guided Cas9 gene‐editing system
Source: Plant Biotechnol J. 2018 May 7;16(11):1892–903. doi: 10.1111/pbi.12924 (PMC6181210; doi:10.1111/pbi.12924)
Supplement: Supplementary file 2 — Appendix S1 Cloning manual for pMGE genome editing vectors. [file PBI-16-1892-s002.pdf]

pMGE vectors for genome editing in monocots [02/2018]

System overview

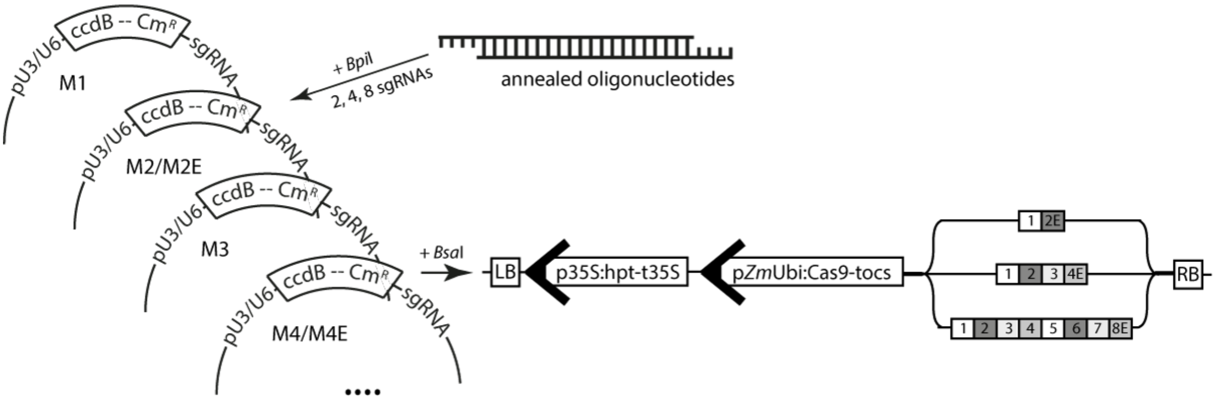

Vector description

name	name (other)	description	resistance [plant]
**recipient (genome editing) vectors**			
pMGE598	pLH_recipient-Hygro	LB_35S:hpt-t35S_pZmUbi:Cas9-tocs; ACTA-BsaI_ccdB-Cm^R_BsaI-GGGA_RB	Spec, Cm [Hygro]
pMGE599	pLH_recipient-Hygro 2xNLS	LB_35S:hpt-t35S_pZmUbi:Cas9(**2xNLS**)-tocs; ACTA-BsaI_ccdB-Cm^R_BsaI-GGGA_RB	Spec, Cm [Hygro]
**sgRNA shuttle vectors – *Oryza sativa* U6 promoter (*Os*U6)**			
pMGE501	pUC M1-OsU6	BsaI-ACTA-link(M13)-pOsU6-GTTG-BpiI_ccdB-Cm^R_BpiI-GTTT-sgRNA scaff.-CGGT-BsaI	Amp, Cm
pMGE503	pUC M2-OsU6	BsaI-CGGT-pOsU6-GTTG-BpiI_ccdB-Cm^R_BpiI-GTTT-sgRNA scaff.-GCAC-BsaI	Amp, Cm
pMGE505	pUC M2E-OsU6	BsaI-CGGT-pOsU6-GTTG-BpiI_ccdB-Cm^R_BpiI-GTTT-sgRNA scaff.-GGGA-BsaI	Amp, Cm
pMGE509	pUC M3-OsU6	BsaI-GCAC-pOsU6-GTTG-BpiI_ccdB-Cm^R_BpiI-GTTT-sgRNA scaff.-AGCG-BsaI	Amp, Cm
pMGE507	pUC M4-OsU6	BsaI-AGCG-pOsU6-GTTG-BpiI_ccdB-Cm^R_BpiI-GTTT-sgRNA scaff.-CCAG-BsaI	Amp, Cm
pMGE508	pUC M4E-OsU6	BsaI-AGCG-pOsU6-GTTG-BpiI_ccdB-Cm^R_BpiI-GTTT-sgRNA scaff.-GGGA-BsaI	Amp, Cm
pMGE516	pUC M1E-OsU6	BsaI-ACTA-link(M13)-pOsU6-GTTG-BpiI_ccdB-Cm^R_BpiI-GTTT-sgRNA scaff.-GGGA-BsaI	Amp, Cm
pMGE512	pUC M5-OsU6	BsaI-CCAG-link(JS838)-pOsU6-GTTG-BpiI_ccdB-Cm^R_BpiI-GTTT-sgRNA scaff.-CAGC-BsaI	Amp, Cm
pMGE513	pUC M6-OsU6	BsaI-CAGC-pOsU6-GTTG-BpiI_ccdB-Cm^R_BpiI-GTTT-sgRNA scaff.-GTTG-BsaI	Amp, Cm
pMGE514	pUC M7-OsU6	BsaI-GTTG-pOsU6-GTTG-BpiI_ccdB-Cm^R_BpiI-GTTT-sgRNA scaff.-CGAA-BsaI	Amp, Cm
pDGE515	pUC M8E-OsU6	BsaI-CGAA-pOsU6-GTTG-BpiI_ccdB-Cm^R_BpiI-GTTT-sgRNA scaff.-GGGA-BsaI	Amp, Cm

**sgRNA shuttle vectors – *Triticum aestivum* U6 promoter (*Ta*U6)**			
pMGE566	pUC M1-TaU6	BsaI-ACTA-link(M13)-pTaU6-CTTG-BpiI_ccdB-Cm^R_BpiI-GTTT-sgRNA scaff.-CGGT-BsaI	Amp, Cm
pMGE567	pUC M2-TaU6	BsaI-CGGT-pTaU6-CTTG-BpiI_ccdB-Cm^R_BpiI-GTTT-sgRNA scaff.-GCAC-BsaI	Amp, Cm
pMGE568	pUC M2E-TaU6	BsaI-CGGT-pTaU6-CTTG-BpiI_ccdB-Cm^R_BpiI-GTTT-sgRNA scaff.-GGGA-BsaI	Amp, Cm
pMGE569	pUC M3-TaU6	BsaI-GCAC-pTaU6-CTTG-BpiI_ccdB-Cm^R_BpiI-GTTT-sgRNA scaff.-AGCG-BsaI	Amp, Cm
pMGE570	pUC M4E-TaU6	BsaI-AGCG-pTaU6-CTTG-BpiI_ccdB-Cm^R_BpiI-GTTT-sgRNA scaff.-GGGA-BsaI	Amp, Cm
pMGE556	pUC M1E-TaU6	BsaI-ACTA-link(M13)-pTaU6-CTTG-BpiI_ccdB-Cm^R_BpiI-GTTT-sgRNA scaff.-GGGA-BsaI	Amp, Cm
**sgRNA shuttle vectors – *Oryza sativa* U3 promoter (*Os*U3)**			
pMGE590	pJ397 M1E-OsU3	BsaI-ACTA-link(M13)-pOsU3-GGCA-BpiI_ccdB-Cm^R_BpiI-GTTT-sgRNA scaff.-GGGA-BsaI	Kan, Cm
pMGE591	pUC M1-OsU3	BsaI-ACTA-link(M13)-pOsU3- GGCA -BpiI_ccdB-Cm^R_BpiI-GTTT-sgRNA scaff.-CGGT-BsaI	Amp, Cm
pMGE592	pUC M2-OsU3	BsaI-CGGT-pOsU3- GGCA -BpiI_ccdB-Cm^R_BpiI-GTTT-sgRNA scaff.-GCAC-BsaI	Amp, Cm
pMGE593	pUC M2E-OsU3	BsaI-CGGT-pOsU3- GGCA -BpiI_ccdB-Cm^R_BpiI-GTTT-sgRNA scaff.-GGGA-BsaI	Amp, Cm
pMGE594	pUC M3-OsU3	BsaI-GCAC-link(JS838)-pOsU3- GGCA -BpiI_ccdB-Cm^R_BpiI-GTTT-sgRNA scaff.-AGCG-BsaI	Amp, Cm
pMGE595	pUC M4E-OsU3	BsaI-AGCG-pOsU3- GGCA -BpiI_ccdB-Cm^R_BpiI-GTTT-sgRNA scaff.-GGGA-BsaI	Amp, Cm
**sgRNA shuttle vectors – *Hordeum vulgare* U3 promoter (*Hv*U3)**			
pMGE584	pJ397 M1E-HvU3	BsaI-ACTA-link(M13)-pHvU3-AGCA-BpiI_ccdB-Cm^R_BpiI-GTTT-sgRNA scaff.-GGGA-BsaI	Kan, Cm
pMGE585	pUC M1-HvU3	BsaI-ACTA-link(M13)-pHvU3-AGCA-BpiI_ccdB-Cm^R_BpiI-GTTT-sgRNA scaff.-CGGT-BsaI	Amp, Cm
pMGE586	pUC M2-HvU3	BsaI-CGGT-pHvU3-AGCA-BpiI_ccdB-Cm^R_BpiI-GTTT-sgRNA scaff.-GCAC-BsaI	Amp, Cm
pMGE587	pUC M2E-HvU3	BsaI-CGGT-pHvU3-AGCA-BpiI_ccdB-Cm^R_BpiI-GTTT-sgRNA scaff.-GGGA-BsaI	Amp, Cm
pMGE588	pUC M3-HvU3	BsaI-GCAC-link(JS838)-pHvU3-AGCA-BpiI_ccdB-Cm^R_BpiI-GTTT-sgRNA scaff.-AGCG-BsaI	Amp, Cm
pMGE589	pUC M4E-HvU3	BsaI-AGCG-pHvU3-AGCA-BpiI_ccdB-Cm^R_BpiI-GTTT-sgRNA scaff.-GGGA-BsaI	Amp, Cm
**sgRNA shuttle vectors – *Hordeum vulgare* U3 promoter (*Hv*U3) with "improved scaffold" (Dang et al., 2015; Genome Biology)**			
pMGE624	pUC M1E-HvU3	BsaI-ACTA-link(M13)-pHvU3-AGCA-BpiI_ccdB-Cm^R_BpiI-GTTT-sgRNA scaff.impr.-GGGA-BsaI	Amp, Cm
pMGE625	pUC M1-HvU3	BsaI-ACTA-link(M13)-pHvU3-AGCA-BpiI_ccdB-Cm^R_BpiI-GTTT-sgRNA scaff.impr.-CGGT-BsaI	Amp, Cm
pMGE626	pUC M2-HvU3	BsaI-CGGT-pHvU3-AGCA-BpiI_ccdB-Cm^R_BpiI-GTTT-sgRNA scaff.impr.-GCAC-BsaI	Amp, Cm
pMGE627	pUC M2E-HvU3	BsaI-CGGT-pHvU3-AGCA-BpiI_ccdB-Cm^R_BpiI-GTTT-sgRNA scaff.impr.-GGGA-BsaI	Amp, Cm
pMGE628	pUC M3-HvU3	BsaI-GCAC-link(JS838)-pHvU3-AGCA-BpiI_ccdB-Cm^R_BpiI-GTTT-sgRNA scaff.impr.-AGCG-BsaI	Amp, Cm
pMGE629	pUC M4E-HvU3	BsaI-AGCG-pHvU3-AGCA-BpiI_ccdB-Cm^R_BpiI-GTTT-sgRNA scaff.impr.-GGGA-BsaI	Amp, Cm

All vectors contain a ccdB cassette and must be propagated in DB3.1 or ccdB survival cells.

shuttle vectors:

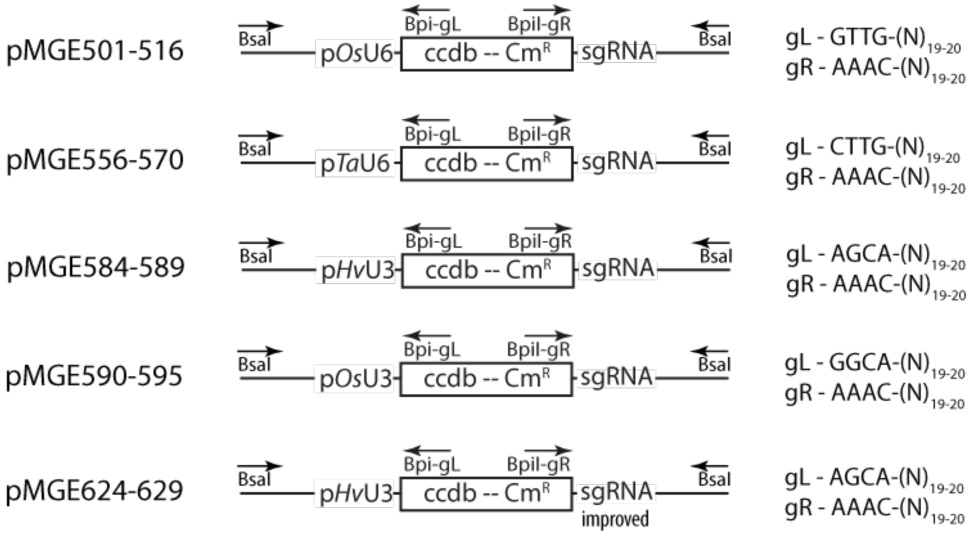

genome editing vectors:

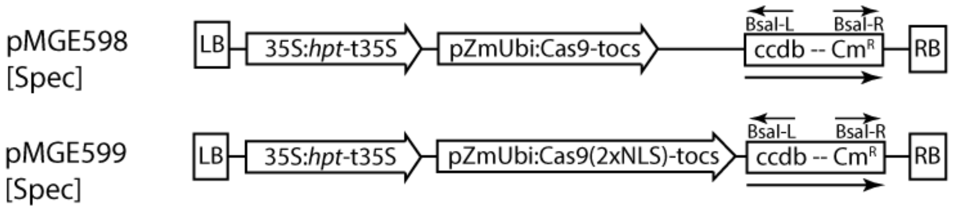

The arrow below the ccdB cassette indicates the direction of the sgRNA array after cloning.

Selection and design of sgRNAs

sgRNA coding sequences are introduced to the pMGE system as 23-24 nt long, hybridized oligonucleotides (oligos). Any PAM sequence (NGG) present in a target region can potentially serve as "anchoring point" for design of an sgRNA, although GC content, on target activity and off-targets should be considered. Note that the PAM sequence is not part of the actual sgRNA, but only present in the target sequence. See for example the following review for a list of tools for selecting target sites: *In Silico* Meets *In Vivo*: Towards Computational CRISPR-Based sgRNA Design (Chuai GH, Wang QL, Liu Q; Trends Biotechnol. 2017 Jan;35(1):12-21. doi: 10.1016/j.tibtech.2016.06.008). Below, examples of (randomly chosen) target sites on plus and minus strands, and design of respective oligos to produce sgRNAs with the pMGE vector system, are given:

sgRNA and oligonucleotide design for U6 promoter shuttle vectors:

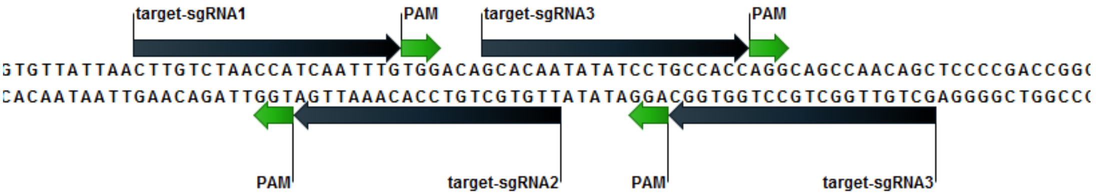

sgRNA1:
Oligo 1 (g/c)ttgCTTGTCTAACCATCAATTTG
Oligo 2 aaacCAAATTGATGGTTAGACAAG

sgRNA2:
Oligo 1 (g/c)ttgTTGTGCTGTCCACAAATTGA
Oligo 2 aaacTCAATTTGTGGACAGCACAA

sgRNA3:
Oligo 1 (g/c)ttGCACAATATATCCTGCCACC
Oligo 2 aaacGGTGGCAGGATATATTGTG

sgRNA4:
Oligo 1 (g/c)ttGCTGTTGGCTGCCTGGTGGC
Oligo 2 aaacGCCACCAGGCAGCCAACAG

Oligo 1 has to start with **g**ttg for **pOsU6** vectors

Oligo 1 has to start with **c**ttg for **pTaU6** vectors

The "G" within the "gttG" or "cttG" used as cloning overhang (Oligo 1) is the transcription start site. sgRNA3 and sgRNA4 are examples for target sites $G(N)_{19}NGG$. In this case, 23 nt oligos may be used, and the variable part of the sgRNA will be 20 nt in length with perfect complementarity. sgRNA1 and sgRNA2 are examples for target sites $(N)_{20}NGG$. In this case, 24 nt oligos should be used. This will result in a sgRNA variable part of 21 nt in length, starting with a non-complementary "G" followed by 20 nt complementary to the target site. To our experience, the "dangling G" when addressing $(N)_{20}NGG$ target sites does not have negative effects on Cas9 efficiency, but this was not evaluated in detail.

sgRNA and oligonucleotide design for U3 promoter shuttle vectors:

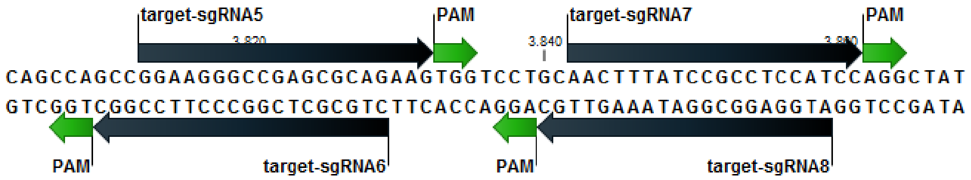

sgRNA5:
Oligo 1 (a/g)gcaGGAAGGGCCGAGCGCAGAAG
Oligo 2 aaacCTTCTGCGCTCGGCCCTTCC

sgRNA6:
Oligo 1 (a/g)gcaCTGCGCTCGGCCCTTCCGGC
Oligo 2 aaacGCCGGAAGGGCCGAGCGCAG

sgRNA7:
Oligo 1 (a/g)gcAACTTTATCCGCCTCCATCC
Oligo 2 aaacGGATGGAGGCGGATAAAGT

sgRNA8:
Oligo 1 (a/g)gcATGGAGGCGGATAAAGTTGC
Oligo 2 aaacGCAACTTTATCCGCCTCCA

Oligo 1 has to start with **a**gca for **pHvU3** vectors

Oligo 1 has to start with **g**gca for **pOsU3** vectors

The "A" within the "agcA" or "ggcA" used as cloning overhang (Oligo 1) is the transcription start site. sgRNA7 and sgRNA8 are examples for target sites $A(N)_{19}NGG$. In this case, 23 nt oligos may be used, and the variable part of the sgRNA will be 20 nt in length with perfect complementarity. sgRNA5 and sgRNA6 are examples for target sites $(N)_{20}NGG$. In this case, 24 nt oligos should be used. This will result in a sgRNA variable part of 21 nt in length, starting with a non-complementary "A" followed by 20 nt complementary to the target site. To our experience, the "dangling A" when addressing $(N)_{20}NGG$ target sites does not have negative effects on Cas9 efficiency, but this was not evaluated in detail.

Not allowed within sgRNA sequences:
BsaI sites [GGTCTC], BpiI sites [GAAGAC], polyT stretches [≥ 5 Ts; transcriptional termination]

Note for sgRNA cloning procedures:
The overhangs used for cloning of hybridized oligos in vectors containing the OsU6 promoter are not ideal. The sticky ends from BpiI digestion (GTTG/GTTT) can re-ligate at a certain frequency, as there is only one nt mismatch. Normally, ~ 10 % of clones on a plate will be the empty vector without the ccdB cassette. Increasing amount of hybridized oligo may help. We advise use of polyclonal plasmid preparations (see below) when working with shuttle vectors.

Cloning and utilization of "one step" vectors

If desired, M1E modules (containing any U3/U6) promoters can be transferred into "recipient" vectors by BsaI cut/ligation to create "one step, one nuclease" vectors as previously described (pDGE62-65 in Ordon et al., 2017, Plant Journal: Generation of chromosomal deletions in dicotyledonous plants employing a user-friendly genome editing toolkit). BsaI cut/ligation reations are essentially prepared as described (page 8), but should be terminated by a final cycle of BsaI digestion (after denaturation) to ligate any remaining "recipient" vector. The following steps should be subsequently conducted to load one step vectors with oligonucleotides to generate functional nucleases:

1. Hybridization of oligos

- oligonucleotide stock concentration: 100 µM
- mix oligos at 10 µM (for example 5 µl of each oligo + 40 µl H_2O)
- denature oligos by heating to 98 °C for 5 min
- let cool down slowly (leaving tube @ RT for several minutes is sufficient)
- prepare a 1:100 dilution (100 fmol / µl) of the hybridized oligos

2. Loading of oligos into recipient vectors

cut/ligation reaction:

20 fmol ≈ 230 ng	pMGE574/575/ 596/ 597
100 fmol = 1 µl	hybridized oligos
1 µl	10 x Ligation buffer
1 µl	10 x BSA (1mg/ml)
0,5 µl	BpiI
0,5 µl	T4 DNA Ligase (1 u/µl)
	H2O
10	Total

37 °C	2 min	10-30 cycles
16 °C	5 min	
50 °C	10 min	
80 °C	10 min	

- transform cut/ligation reaction into Dh10b/TopTen cells
- plate on LB-Spec media
- start liquid cultures from 1-2 colonies for plasmid preparation
- sequence sgRNA fraction (Primer JS1057)
- transform into your favorite *Agrobacterium* strain

Cloning of multiplex genome editing constructs (pMGE598, pMGE599)

The cloning of multiplex genome editing constructs necessitates first the loading of sgRNA shuttle vectors with hybridized oligos by BpiI cut/ligation to generate the desired sgRNA transcriptional units (sgRNA TUs). Subsequently, 2, 4, or 8 sgRNA TUs are assembled in a genome editing vector by BsaI cut/ligation to generate a final construct for plant transformation. Modules are named according to their position within the final sgRNA array, and modules for ending the array (circularization of the vector) are named with an additional "E". Modules containing different U3/U6 promoters may be combined, if desired. In the following, compatible combinations using only one type of U3/U6 promoter are listed:

number of sgRNA TUs	Pol III promoter	derivatives of pMGE for sgRNA array assembly
2 sgRNA TUs	OsU6	pMGE501, 505
	TaU6	pMGE566, 568
	HvU3	pMGE585, 587
	OsU3	pMGE591, 593
	HvU3 (impr. sgRNA scaffold)	pMGE625, 627
4 sgRNA TUs	OsU6	pMGE501, 503, 509, 508
	TaU6	pMGE566, 567, 569, 570
	HvU3	pMGE585, 586, 588, 589
	OsU3	pMGE591, 592, 594, 595
	HvU3 (impr. sgRNA scaffold)	pMGE625, 626, 628, 629
8 sgRNA TUs	OsU6	pMGE501, 503, 509, 507, 512, 513, 514, 515

1. Hybridization of oligos
   - oligo stock concentration: 100 µM
   - mix oligos at 10 µM (for example 5 µl of each oligo + 40 µl H_2O)
   - denature oligos by heating to 98 °C for 5 min
   - let cool down slowly (leaving tube @ RT for several minutes is sufficient)
   - prepare a 1:100 dilution (100 fmol / µl) of the hybridized oligos

2. Loading of oligos = guide sequences into sgRNA shuttle vectors

cut/ligation reaction:

20 fmol ≈ 60 ng	shuttle vector
100 fmol = 1 µl	hybridized oligos
1 µl	10 x Ligation buffer
1 µl	10 x BSA (1mg/ml)
0,5 µl	BpiI
0,5 µl	T4 DNA Ligase (1 u/µl)
	H_2O
10	Total

37 °C	2 min	10-30 cycles
16 °C	5 min	
50 °C	10 min	
80 °C	10 min	

monoclonal approach:

- transform cut/ligation reaction into Dh10b/TopTen cells
- plate on selective media (Amp)
- start liquid cultures from 1-2 colonies for plasmid preparation
- sequence using M13r primer
  [Do not use M13f for M1/M1E modules]

polyclonal approach **(adviced)**:

- transform cut/ligation reaction into Dh10b/TopTen cells
- directly inoculate liquid cultures with transformed cells in selective media
- use liquid cultures for polyclonal plasmid preparation

3. Assembly of sgRNA TUs in genome editing vector

cut/ligation reaction:

20 fmol ≈ 230 ng	recipient vector
20 fmol ≈ 40 ng	sgRNA TU shuttle vectors
2 µl	10 x Ligation buffer
2 µl	10 x BSA (1mg/ml)
1 µl	BsaI
1 µl	T4 DNA Ligase (1-5 u/µl)
	H_2O
20	Total

37 °C	2 min	30-50 cycles
16 °C	5 min	
50 °C	10 min	
80 °C	10 min	

- transform cut/ligation reaction into Dh10b/TopTen cells
- plate on selective media (Spec)
- 2 sgRNA TUs: Start liquid cultures from 2 clones
- 4 sgRNA TUs: Start liquid cultures from 2-4 clones
- 8 sgRNA TUs: Start liquid cultures of 4-6 colonies directly or do colony PCR on 8-16 clones.
- verify plasmids by restriction digest
- confirm sgRNA array by DNA sequencing (Primers JS1057 and/or M13f)
- transform plasmid into your favorite *Agrobacterium* strain

Previously used oligos for DNA sequencing:

name	sequence	location
**M13f**	GTTTTCCCAGTCACGAC	sgRNA TU 1, fwd
**JS838**	GCCAGCTTCTATGAGTACTGA	sgRNA TU 3 or 5, fwd
**JS1057**	CATCAGACAAACCGGCCAG	vector, rev

Additional oligos can be designed in linker sequences included in shuttle vectors.
